# Axial and Asymmetric Coordination Coupling Adjust the Electronic Structure of Single‐Atom Zinc Sites for Efficient Electroreduction of Carbon Dioxide

**DOI:** 10.1002/advs.202509698

**Published:** 2025-09-14

**Authors:** Cao Guo, Feng Wang, Abdukader Abdukayum, Qingde Chen, Fengqin Chang, Hongyi Li, Xuguang An, Guangzhi Hu, Yujie Ma

**Affiliations:** ^1^ Xinjiang Key Laboratory of Novel Functional Materials Chemistry College of Chemistry and Environmental Sciences Kashi University Kashi 844000 China; ^2^ Qilu Lake Field Scientific Observation and Research Station for Plateau Shallow Lake in Yunnan Province Institute for Ecological Research and Pollution Control of Plateau Lakes School of Ecology and Environmental Science Yunnan University Kunming 650504 China; ^3^ State Key Laboratory of Chemistry and Utilization of Carbon Based Energy Resources College of Chemistry Xinjiang University Urumqi 830017 China; ^4^ School of Mechanical Engineering Chengdu University Chengdu 610106 China; ^5^ Department of Chemistry University of Manchester Manchester M13 9PL UK

**Keywords:** asymmetric coordination, axial coordination, CO_2_ electroreduction, electronic modulation, single‐atom catalysts

## Abstract

Breaking the symmetric structure of active centers to adjust their electronic structure is a promising strategy for improving the performance of single‐atom catalysts (SACs) in electrocatalytic carbon dioxide (CO_2_) reduction (ECR). However, it remains highly challenging to achieve precise regulation and fine‐tuning of single‐atom sites at the atomic level. Here, by introducing S and Cl atoms, a Zn‐SAC (ZnN_3_S_1_Cl/C) with coupled axial and asymmetric coordination is successfully constructed, thereby enhancing the ECR performance. In situ attenuated total reflection infrared spectroscopy demonstrates that ZnN_3_S_1_Cl/C promotes the formation of ^*^COOH and the desorption of ^*^CO species. Theoretical calculations show that the asymmetric coordination of S and the axial coordination of Cl can lead to the electron redistribution near the single Zn sites, increasing the overlap between the Zn (3d) and ^*^COOH (2p) orbitals. This enhances the adsorption strength of ^*^COOH on the Zn site and reduces the desorption energy of ^*^CO, thus facilitating catalytic performance. Therefore, the ZnN_3_S_1_Cl/C catalyst achieves a CO faradaic efficiency of ≈100% in an H‐cell, with excellent long‐term stability of 240 h. This work may pave the way for the development of efficient ECR catalysts via fine manipulation of asymmetric and electronic structures of single‐atom metal sites.

## Graphical abstract

Zn‐SACs are successfully synthesized with axial Cl and asymmetric S coordination (ZnN_3_S_1_Cl/C). The asymmetric coordination of S and the axial coordination of Cl can lead to electron redistribution near the single Zn site, increasing the overlap between the Zn (3d) and ^*^COOH (2p) orbitals. This orbital overlap enhances the adsorption strength of ^*^COOH on the Zn site and helps reduce the desorption energy of ^*^CO. Therefore, the ZnN_3_S_1_Cl/C catalyst achieves a CO Faradaic efficiency (FE_CO_) of ≈100%.

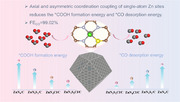

## 1 Introduction

Breaking the natural carbon cycle has led to an annual increase in the atmospheric CO_2_ concentration, resulting in global warming and a series of environmental issues, including droughts, floods, and a rise in sea levels.^[^ ^1^ ^]^ To address these challenges, new technologies aimed at carbon capture and utilization are being explored. The use of clean, renewable electricity to drive electrocatalytic CO_2_ reduction (ECR) represents a promising pathway for developing suitable technologies to convert CO_2_ into chemicals and fuels for reducing carbon emissions.^[^ ^2^ ^]^ Furthermore, considering the energy input required for the ECR process, the subsequent separation and purification costs of the products, and the application prospects of CO, converting CO_2_ into CO represents an economically viable pathway.^[^ ^3^ ^]^ However, the overlap of the reaction potentials of the ECR and hydrogen evolution reaction (HER) leads to unsatisfactory Faradaic efficiency (FE_CO_) and energy efficiency of CO production, which typically involves multiple proton‐coupled electron‐transfer steps, resulting in poor reaction kinetics.^[^ ^4^ ^]^ Therefore, the feasibility of the process ultimately depends on the development of competitive catalysts that can meet the operational requirements of the catalytic activity, selectivity, and stability.^[^ ^5^ ^]^

In recent years, the research focus in the field of ECR for CO production has been on single‐atom catalysts, which feature a low metal loading and yet exhibit a high catalytic activity, with the theoretical atom utilization approaching 100%.^[^ ^6^ ^]^ Owing to the significant reduction in the particle size, resulting in a high surface free energy, the isolated metal atoms of SACs typically coordinate with the adjacent atoms on the support surface.^[^ ^7^ ^]^ Consequently, the local atomic environment of SACs is a decisive factor that determines the performance of the catalyst.^[^ ^8^ ^]^ Currently, the extensively studied SACs for the ECR to CO primarily feature porphyrin‐like planar M‐N_4_ coordination structures (where M represents a metal). The uniqueness of such structures has been fully demonstrated in the field of ECR.^[^ ^9^ ^]^ However, recent studies have indicated that the symmetric electron distribution on nonpolar M‐N_4_ moieties restricts the activity of the central metal atom, potentially leading to nonoptimal adsorption strengths of the reaction intermediates, ultimately resulting in slow ECR kinetics.^[^ ^10^ ^]^ Clearly, the intrinsic activities exhibited by the SACs with symmetric M‐N_4_ configurations still deviate from the peak of the volcano plot. The key to further enhancing the performance of M‐N_4_ catalysts lies in breaking the symmetric coordination to optimise their electronic structure, which is crucial for boosting their catalytic activity/selectivity. This is because an asymmetric coordination structure enables the metal site to accommodate as many electrons as possible to stabilise the reaction intermediates and facilitate bond breakage and reformation.^[^ ^11^ ^]^

Generally, an asymmetric arrangement of electrons can be achieved by adjusting the number of coordinating N atoms around the central M atom and/or by introducing nonmetallic heteroatoms with different electronegativities and atomic radii. For instance, Li et al. fabricated a Zn‐based SAC with unsaturated ZnN_3_ coordination.^[^ ^12^ ^]^ Compared with the Zn atom in ZnN_4_, the electron‐rich environment of the Zn atom in ZnN_3_ facilitated the stabilization of the ^*^COOH intermediate, significantly reducing the energy barrier of the reaction. Wang et al. constructed an SAC featuring asymmetric Ca‐N_3_O coordination using *p*‐block metals and demonstrated that the establishment of asymmetric coordination improved the electron localization at the Ca centre, enhancing the dynamics of electron transfer.^[^ ^11a^ ^]^ Although these strategies have proven to be viable for enhancing the activity of single‐atom centres and improving the ECR performance for CO production, significant challenges remain in terms of precisely tuning the coordination environment of the metal centre, particularly through the incorporation of multiple heteroatoms to modulate its electronic structure.^[^ ^13^ ^]^ Furthermore, there is a lack of robust mechanistic insights that explain how the changes in single‐atom coordination structures affect the electrocatalytic performance of SACs.^[^ ^14^ ^]^ Therefore, there is an urgent need for atomic‐level regulation of the coordination environment of SACs and a comprehensive elucidation of how the changes in the electronic structure of SACs enhance the ECR kinetics.

In this study, we altered the symmetric coordination structure of ZnN_4_ by coordinating it with S and Cl atoms. Specifically, we successfully synthesised Zn SACs with axial Cl and asymmetric S coordination (ZnN_3_S_1_Cl/C) using a simple liquid‐phase deposition method followed by one‐step pyrolysis. The purpose of constructing this SAC configuration was to break the symmetric coordination of ZnN_4_ and thus modify the electronic structure of the Zn centre to realise an efficient ECR to CO. Various electron microscopy and spectroscopy techniques confirmed the single‐atom nature of the Zn centre and its coordination with N, S, and Cl atoms. Moreover, theoretical calculations supported the stable presence of these atoms in the modified catalyst and the precise control achieved over the single‐atom coordination environment. The change in the Zn‐centre coordination led to a markedly improved ECR performance. ZnN_3_S_1_Cl/C achieved ≈100% FE_CO_ and long‐term stability (> 240 h of operation) in an H‐cell, with negligible attenuation of FE_CO_. Furthermore, in a flow cell, ZnN_3_S_1_Cl/C achieved ≈90% FE_CO_ at a current density of ≈50 mA cm^−2^, confirming its practical application potential. Further, the CO_2_ electroreduction processes over the ZnN_3_S_1_Cl/C and ZnN_4_/C catalysts were investigated using in situ attenuated total reflection infrared (ATR‐IR) spectroscopy. The ATR‐IR results revealed that the coupling of axial and asymmetric coordination promoted the formation of the ^*^COOH intermediate at the active site and the subsequent desorption of ^*^CO from the catalyst surface. Furthermore, theoretical calculations supported that the ZnN_3_S_1_Cl/C configuration not only favours the desorption of ^*^CO but also enhances the adsorption strength of ^*^COOH on the Zn site; the latter is due to increased overlap between the Zn (3d) and ^*^COOH (2p) orbitals. This orbital overlap lowered the energy barrier for ^*^COOH formation, thereby providing a better ECR performance. This study not only provides important guidelines for the design of CO_2_ electrocatalytic reduction catalysts but also clarifies the intrinsic active sites and catalytic pathways at the atomic level.

## 2 Results and Discussion

**Figure 1:**
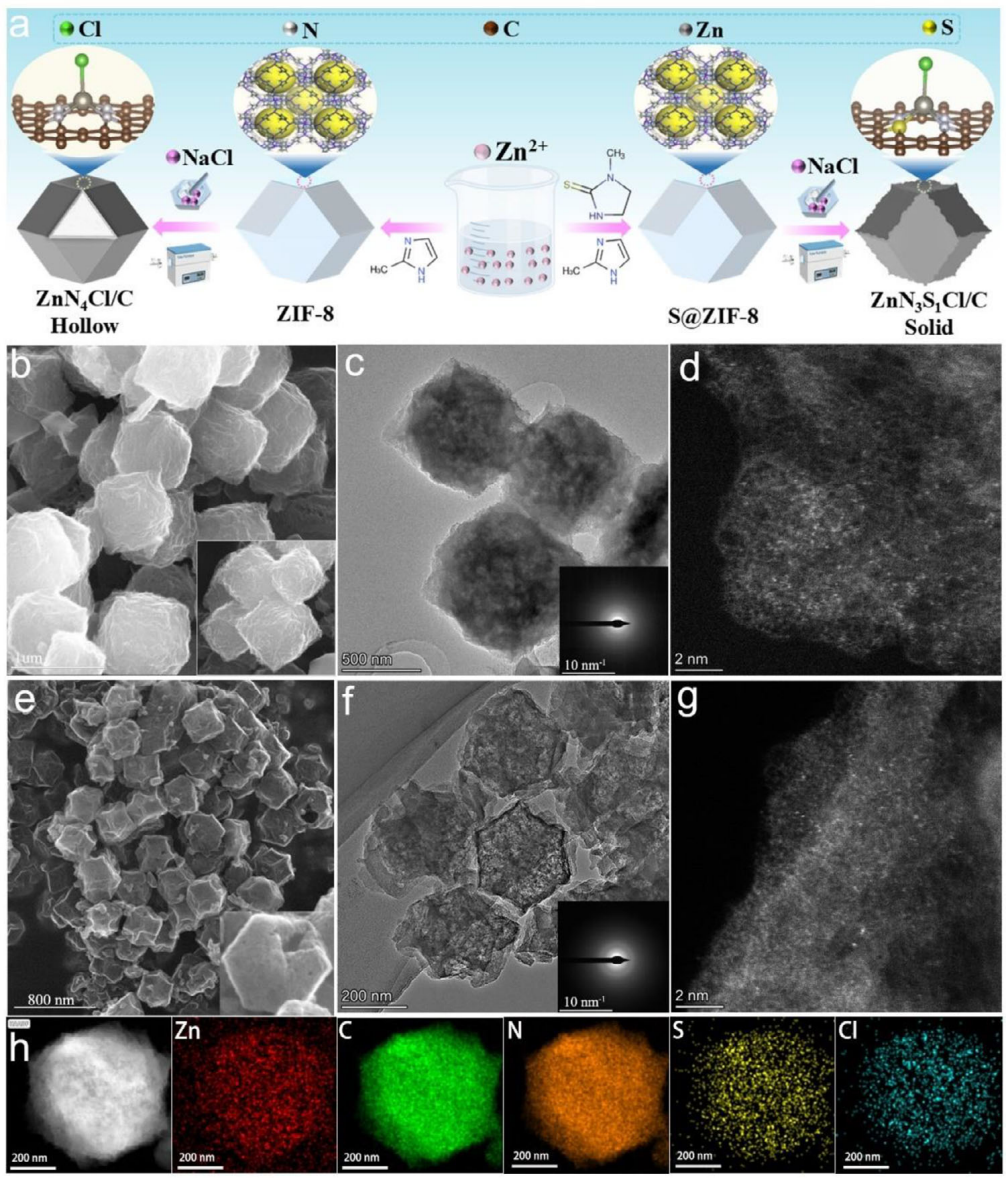
a) Preparation process of ZnN_4_Cl/C and ZnN_3_S_1_Cl/C catalysts. b) SEM and c) TEM (inset showing SAED) images of ZnN_3_S_1_Cl/C. d) AC HAADF‐STEM image of ZnN_3_S_1_Cl/C. e) SEM and f) TEM (inset showing SAED) images of ZnN_4_Cl/C. g) AC HAADF‐STEM image of ZnN_4_Cl/C. h) HAADF‐STEM image and corresponding EDS element mappings of ZnN_3_S_1_Cl/C.

Two samples, viz., ZnN_3_S_1_Cl/C as the target and ZnN_4_Cl/C as a control sample, were prepared using a two‐step method, as illustrated in **Figure** **1**a. First, the one‐pot synthesis of ZIF‐8 containing mercapto‐groups (denoted as S@ZIF‐8) was conducted using dimethylimidazole and mercaptoimidazole as mixed ligands. When only dimethylimidazole was used as the ligand, ZIF‐8 without mercapto groups was obtained. Raman spectroscopy revealed that S@ZIF‐8 yields a distinct peak at ∼2450 cm^−1^ that can be attributed to the vibration of the ─SH group,^[^ ^15^ ^]^ indicating the successful incorporation of mercapto groups (Figure S1, Supporting Information). Scanning electron microscopy (SEM) images of ZIF‐8 and S@ZIF‐8 revealed that both samples had a dodecahedral structure with uniform sizes. The average particle diameters of ZIF‐8 and S@ZIF‐8 were however, different at 400 nm and 1 µm (Figure S2, Supporting Information), respectively.

Next, to incorporate Cl, the ZIF‐8 and S@ZIF‐8 samples were individually mixed with NaCl and milled. The mixtures were then pyrolysed at 1000 °C and washed with water to remove residual NaCl, recovering black powders designated as ZnN_4_Cl/C and ZnN_3_S_1_Cl/C, respectively. During the pyrolysis of these samples, in addition to the partial volatilization of Zn atoms and the embedment of Cl atoms, the precursor framework was etched away by molten NaCl. Owing to the greater chemical stability of the outer layers compared with that of the interior of metal‐organic frameworks,^[^ ^16^ ^]^ the inner portion of ZIF‐8 was preferentially etched by NaCl, leading to the formation of a hollow ZnN_4_Cl/C structure (Figure 1e). However, the larger size of the S@ZIF‐8 MOF protected its inner layers, resulting in the formation of ZnN_3_S_1_Cl/C with a rough surface, rather than a hollow ZnN_3_S_1_Cl/C structure, due to etching by molten NaCl (Figure 1b). Transmission electron microscopy (TEM) images revealed that no metal particles or compounds were formed in either the solid ZnN_3_S_1_Cl/C particles or the hollow ZnN_4_Cl/C structures (Figure 1c,f). The selected area electron diffraction (SAED) patterns of the two samples showed diffraction rings attributed to carbon, and no metallic crystalline substances were detected (see insets in Figure 1c,f). Additionally, two control samples, viz., ZnN_4_/C and ZnN_3_S_1_/C, were also prepared by the direct calcination of ZIF‐8 and S@ZIF‐8. Both ZnN_4_/C and ZnN_3_S_1_/C retained the dodecahedral morphology of the precursors, without the formation of metal aggregates, and exhibited a uniform elemental distribution (Figures S4 and S5, Supporting Information). The powder X‐ray diffraction patterns of all four catalysts exhibited only two broad peaks corresponding to graphitic carbon, with no peaks associated with metal aggregates (Figure S6, Supporting Information), consistent with the TEM results.

Aberration‐corrected high‐angle annular dark‐field scanning transmission electron microscopy (AC HAADF‐STEM) images in Figure 1d,g confirmed the presence of isolated Zn sites and the absence of Zn nanoparticles in the ZnN_3_S_1_Cl/C and ZnN_4_Cl/C samples. The corresponding elemental mapping images indicate the uniform distribution of Zn, C, N, S, and Cl throughout the framework (Figures 1h; S7, Supporting Information). The contents of Zn in ZnN_3_S_1_Cl/C, ZnN_4_Cl/C, ZnN_3_S_1_/C, and ZnN_4_/C, as determined by inductively coupled plasma mass spectrometry (ICP‐MS), were 1.09, 1.05, 1.12, and 1.35 wt%, respectively. According to N_2_ adsorption/desorption experiments, the Brunauer–Emmett–Teller (BET) surface areas of the sulfur‐containing ZnN_3_S_1_Cl/C and ZnN_3_S_1_/C catalysts were 1389 and 1252 m^2^ g^−1^, respectively (Figure S8, Supporting Information), while those of the ZnN_4_Cl/C and ZnN_4_/C were 1772 and 1535 m^2^ g^−1^, respectively (Figure S9, Supporting Information). These findings further prove that the NaCl‐melt‐etching of the MOFs leads to either a roughened surface or hollow morphology. These structural changes are advantageous for increasing the specific surface area of the catalysts, as they facilitate a higher exposure of the active Zn sites and improve the mass transfer capability.^[^ ^17^ ^]^ Additionally, the Raman spectra of the four catalysts primarily exhibited the characteristic D and G bands of graphitic carbon (Figure S10, Supporting Information). An increased intensity ratio of the D to G bands (*I*_D_/*I*_G_) typically indicates a higher degree of defects and disorder. The *I*_D_/*I*_G_ ratios of the samples suggested that ZnN_3_S_1_Cl/C has a higher level of defects than the other samples. Overall, the etching effect of NaCl is beneficial for increasing the specific surface area and defect density of the catalyst, which may facilitate the catalytic performance.

**Figure 2:**
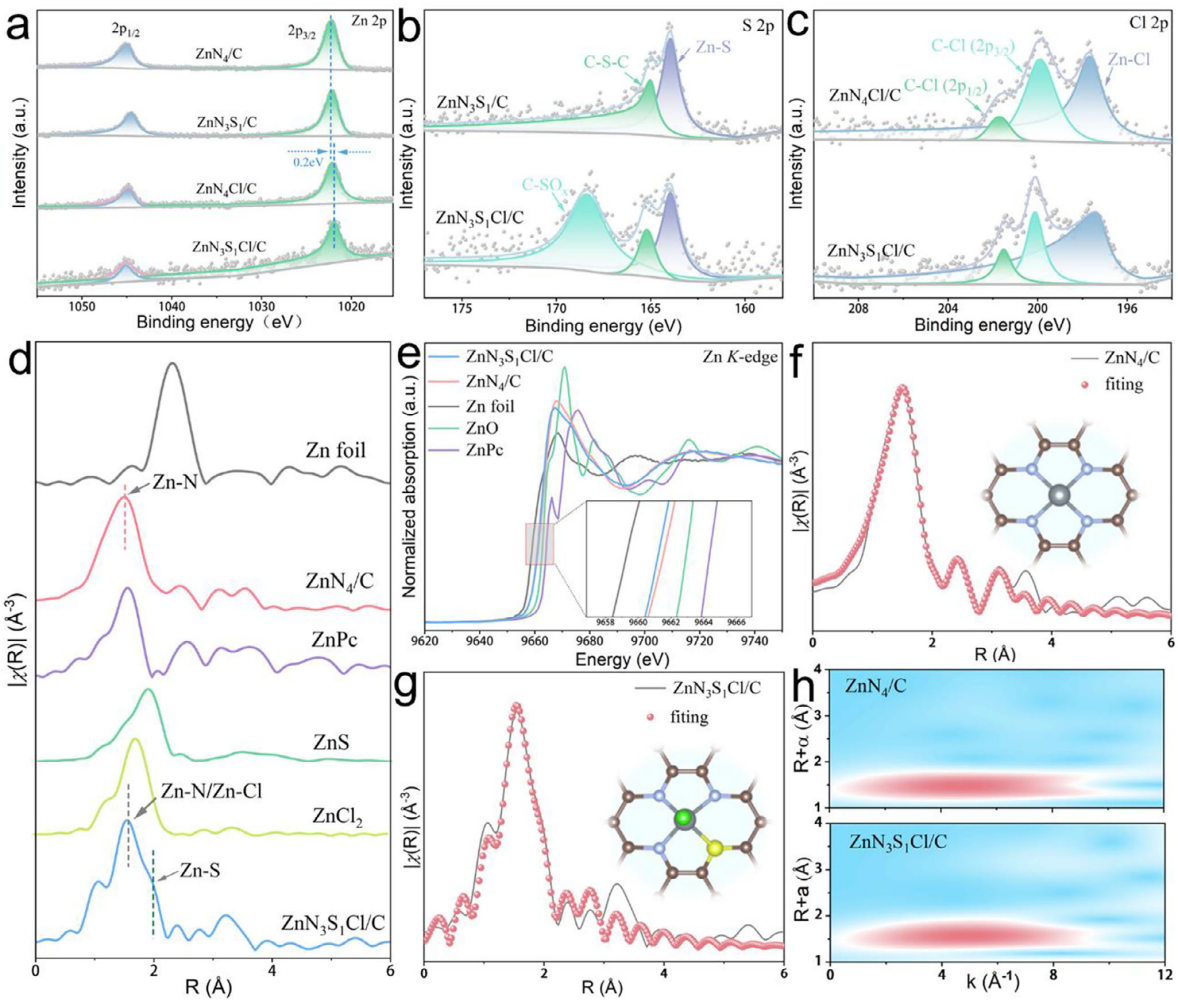
(a) Zn 2p, (b) S 2p, and (c) Cl 2p XPS spectra of different catalysts. (d) Fourier transform of the Zn *K*‐edge EXAFS spectra and (e) Zn *K*‐edge XANES spectra of ZnN_4_/C, ZnN_3_S_1_Cl/C, and the references. Zn *K*‐edge EXAFS fitting results of (f) ZnN_4_/C and (g) ZnN_3_S_1_Cl/C (inset showing the coordination structure of the Zn single‐atom). (h) WT‐EXAFS of ZnN_4_/C and ZnN_3_S_1_Cl/C.

X‐ray photoelectron spectroscopy (XPS) and X‐ray absorption spectroscopy (XAS) were used to further investigate the chemical valence states and coordination configurations of the elements in the four catalysts. The C 1s XPS profile of ZnN_3_S_1_Cl/C (Figure S11a, Supporting Information) exhibited four peaks attributed to C═C (284.8 eV), C─S (286.3 eV), C─Cl (288.7 eV), and C─N (290.6 eV).^[^ ^18^ ^]^ The N 1s XPS profiles of the four catalysts displayed similar peaks corresponding to oxidised N, graphitic N, Zn‐N, and pyridinic N (Figure S11b, Supporting Information).^[^ ^19^ ^]^ The Zn 2p XPS profiles of the samples in **Figure** **2**a exhibit peaks at ∼1021.9 and 1045.1 eV attributed to Zn 2p_1/2_ and Zn 2p_3/2_, respectively. The peak at 1021.9 eV lies between 1022.8 eV (Zn^2+^) and 1021.6 eV (Zn^0^), indicating that the valence state of Zn in ZnN_3_S_1_Cl/C is between +2 and 0.^[^ ^20a^ ^]^ Additionally, compared with that of ZnN_4_/C, the Zn 2p_3/2_ peak of ZnN_3_S_1_Cl/C was shifted by ∼0.2 eV toward the lower binding‐energy region, suggesting that after the introduction of S and Cl heteroatoms, some electrons may be transferred to Zn through S and Cl.^[^ ^20^ ^]^ This electron transfer may lead to a negative shift of the *d*‐band centre of the Zn atom relative to the Fermi level, potentially enhancing electron localization.^[^ ^21^ ^]^ The S 2p spectrum of ZnN_3_S_1_Cl/C (Figure 2b) presents three peaks at 163.9, 165.2, and 168.4 eV, which are attributed to Zn─S, C─S─C, and C─SO_x_ (sulfate species),^[^ ^19a^ ^]^ respectively. Further, the Cl 2p XPS profiles of ZnN_4_Cl/C and ZnN_3_S_1_Cl/C exhibit a peak corresponding to Zn─Cl at ∼197.4 eV and two more peaks attributable to Cl─C bonding at ∼200.1 (2p_3/2_) and ∼201.5 eV (2p_1/2_) (Figure 2c).^[^ ^19^, ^22^ ^]^ Thus, XPS confirmed that N, S, and Cl were successfully introduced into the ZnN_3_S_1_Cl/C catalyst and that they were coordinated to the Zn atoms.

To precisely understand how the coordination geometry of Zn atoms changes in the catalyst after the introduction of S and Cl heteroatoms, Fourier transform (FT) extended X‐ray absorption fine structure (EXAFS) analysis was conducted. As shown in Figure 2d, the main peak in the EXAFS profile of ZnN_4_/C is located at 1.50 Å, which is close to the Zn‐N peak (1.53 Å) of ZnPc; therefore, it is attributed to Zn─N coordination in ZnN_4_/C. For ZnN_3_S_1_Cl/C, the main peak is located at 1.56 Å, which falls between the Zn─N peak of ZnPc and the Zn─Cl peak (1.71 Å) of ZnCl_2_; thus, it corresponds to the Zn─N/Zn─Cl coordination in ZnN_3_S_1_Cl/C. Additionally, a shoulder peak is observed at 1.90 Å in the Zn K‐edge EXAFS spectrum of ZnN_3_S_1_Cl/C, which is close to the Zn─S peak (1.88 Å) of ZnS; thus, it is attributed to Zn─S coordination in ZnN_3_S_1_Cl/C. EXAFS spectra of ZnN_4_/C and ZnN_3_S_1_Cl/C (Figure 2d) show a lower intensity for the features at a long distance (≈2.3 Å) in the Fourier transform of the k^2^‐weighted data compared with that for Zn foil as a reference material. This strongly suggests that the Zn sites in both samples are atomically dispersed, consistent with the HAADF‐STEM results. The Zn K‐edge X‐ray absorption near‐edge structure (XANES) spectra (Figure 2e) revealed that the absorption edges of ZnN_4_/C and ZnN_3_S_1_Cl/C lie between those of Zn foil and ZnO, indicating that the valence states of Zn in ZnN_4_/C and ZnN_3_S_1_Cl/C are between 0 and +2. Importantly, ZnN_3_S_1_Cl/C exhibited a lower peak intensity than ZnN_4_/C (inset in Figure 2e), indicating that the introduction of Cl and S reduced the oxidation state of Zn. By further fitting the edge energy, the average valence of Zn in the ZnN_4_/C and ZnN_3_S_1_Cl/C is calculated to be 1.16 and 1.01 (Figure S12, Supporting Information).^[^ ^23a,b^ ^]^ Overall, the coordination and valence states determined by EXAFS and EANES are consistent with the XPS results. The aforementioned results also indicate that the introduction of Zn─S and Zn─Cl coordination elongates the Zn─N bond length in ZnN_3_S_1_Cl/C, reduces the valence state of Zn, and enhances the stability of the catalyst. Additionally, the specific coordination states of the Zn sites in ZnN_4_/C and ZnN_3_S_1_Cl/C were determined through quantitative least‐squares EXAFS curve fitting. The optimal fitting results indicated that each Zn atom in ZnN_4_/C was coordinated with four N atoms, whereas in ZnN_3_S_1_Cl/C, the Zn atom was coordinated with three N atoms and S and Cl atoms (Figure 2f,g; Table S1, Supporting Information). The maximum intensities in the wavelet transform (WT)‐EXAFS contour plots of ZnN_4_/C and ZnN_3_S_1_Cl/C were observed at ≈4.8 Å (Figure 2h), which is a typical feature of atomically dispersed Zn.^[^ ^18^, ^20^ ^]^ Overall, the single‐atom nature of the Zn sites in catalysts is confirmed by combining HAADF‐STEM and XAS results.

**Figure 3:**
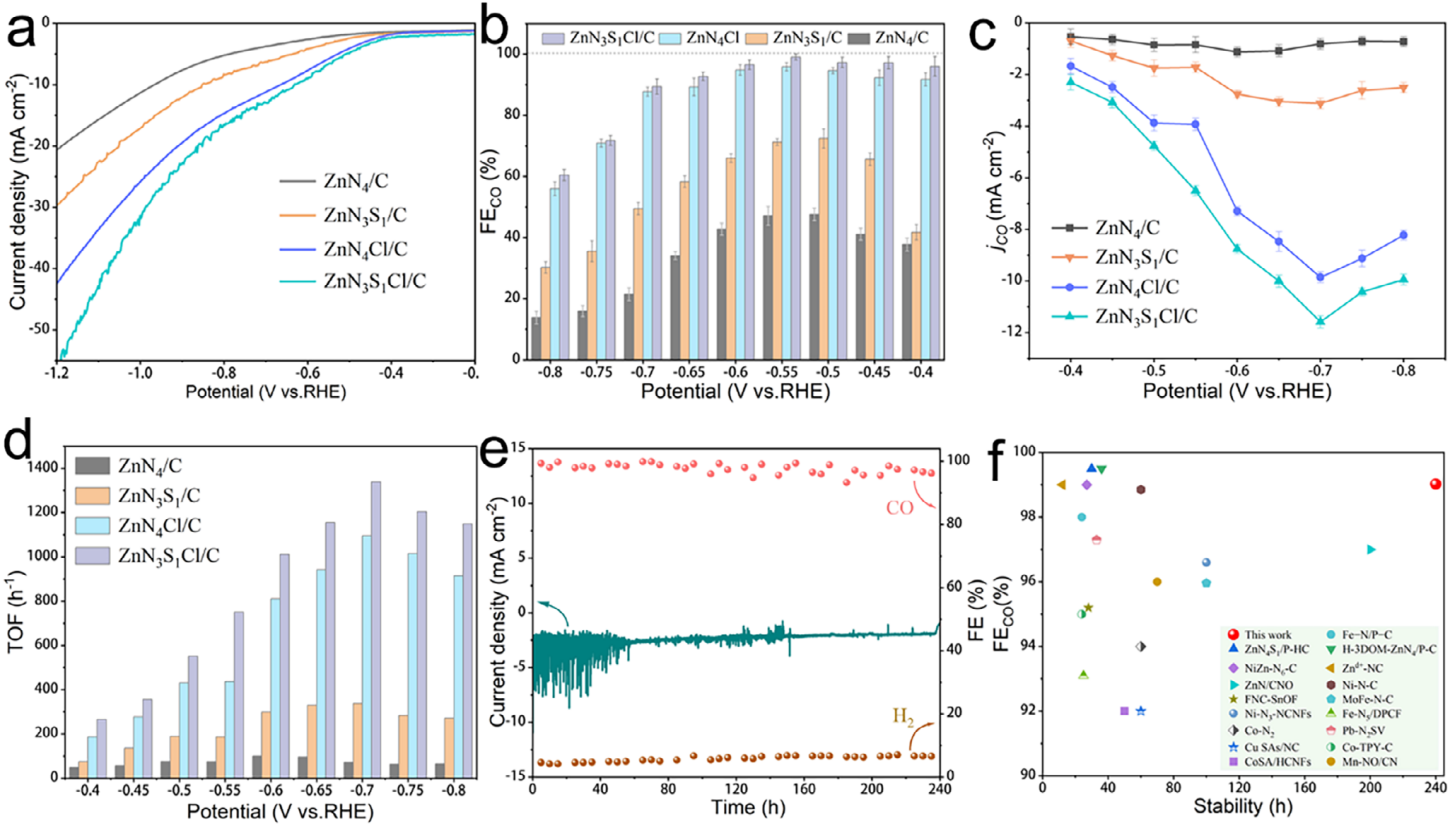
ECR performance of the catalysts. a) LSV curves acquired in H‐Cell. b) FE_CO_, c) *j*_CO_ and d) TOF of the catalysts. e) Stability test at −0.55 V over the ZnN_3_S_1_Cl/C. f) ECR performance of the ZnN_3_S_1_Cl/C compared with those of reported single‐atom catalysts for CO production.

To preliminarily evaluate the ECR performance of the catalysts, electrochemical tests were conducted in an H‐cell using 0.5 M KHCO_3_ as the electrolyte (Figure S13, Supporting Information). The linear sweep voltammetry (LSV) curves of the samples (**Figures** **3**a; S14, Supporting Information) reveal that ZnN_3_S_1_Cl/C has a higher current density, indicating that the introduction of S and Cl atoms enhanced the electrochemical activity of the system. When the ECR products were analysed by gas chromatography (GC), only CO and H_2_ were detected as gaseous products (Figure S15, Supporting Information). The total Faradaic efficiency was close to 100%, and the onset potential of the ECR on ZnN_3_S_1_Cl/C was lower than those of the control samples (Figure S16, Supporting Information). The FE_CO_ of ZnN_3_S_1_Cl/C was >90% over a wide potential range of −0.4–−0.7 V versus reversible hydrogen electrode (versus RHE, all potentials hereafter are referenced to RHE), and the maximum FE_CO_ of 99.02% was achieved at −0.55 V (Figure 3b).

At the same potential, the ZnN_3_S_1_Cl/C catalyst exhibited a higher CO partial current density (*j*_CO_) and turnover frequency (TOF) for CO production per metal site compared with those of the control samples (Figure 3c,d). At −0.7 V, the *j*_CO_ and TOF of ZnN_3_S_1_Cl/C were 11.6 mA cm^−2^ and 1338 h^−1^, which are 14.3 and 18.5 times higher than those of ZnN_4_/C, respectively. These results indicate that breaking the symmetric coordination structure of the single Zn atom in the ZnN_3_ catalyst is beneficial for modulating its catalytic activity. Apart from the considerable ECR activity, ZnN_3_S_1_Cl/C also exhibited excellent durability in an H‐cell. After continuous operation at −0.55 V for 240 h, the FE_CO_ decay was <5%, that is, the FE_CO_ remained above 95% (Figure 3e). After stability test, little difference was observed in the XRD patterns, XPS spectra or Raman data for fresh and used samples (Figures S18–S20, Supporting Information), confirming the stability of the catalyst. The excellent FE_CO_ and long‐term stability of ZnN_3_S_1_Cl/C surpass those of most of the reported SACs (Figure 3f; Table S2, Supporting Information). In particular, almost all the previously reported single‐atom, diatom, and cluster catalysts lose activity after 200 h, confirming the excellent durability of ZnN_3_S_1_Cl/C.

The origin of the enhanced catalytic performance of ZnN_3_S_1_Cl/C was further investigated through Tafel analyses. The Tafel slope of ZnN_3_S_1_Cl/C was found to be 124.6 mV dec^−1^ (Figure S21, Supporting Information), significantly lower than those of ZnN_4_Cl/C (201.9 mV dec^−1^), ZnN_3_S_1_/C (308.9 mV dec^−1^), and ZnN_4_/C (383.8 mV dec^−1^). The Tafel slope generally represents the first electron transfer in the reaction and represents the rate‐limiting step of the overall reaction process.^[^ ^24^ ^]^ The lower Tafel slope of the ZnN_3_S_1_Cl/C catalyst indicates that the generation of ^*^COOH intermediate is facilitated on the catalyst surface. Electrochemical impedance analysis revealed that ZnN_3_S_1_Cl/C had the smallest charge‐transfer resistance, indicating its superior reaction kinetics (Figure S22, Supporting Information). In addition, the double‐layer capacitance (*C*_dl_) of ZnN_3_S_1_Cl/C was determined to be 16.9 mF cm^−2^, greater than those of ZnN_4_Cl/C (9.1 mF cm^−2^), ZnN_3_S_1_/C (8.4 mF cm^−2^), and ZnN_4_/C (5.3 mF cm^−2^), indicating that the ZnN_3_S_1_Cl/C catalyst has a larger electrochemical active surface area (Figures S23 and S24, Supporting Information). Furthermore, to demonstrate that Zn with axial and asymmetric coordination is the main source of catalyst activity, we conducted SCN^−^ ion poisoning experiments. SCN^−^ is a powerful field ligand that readily forms coordination compounds with metal ions, thereby hindering the interaction between metal sites and CO_2_. After introducing SCN^−^ into the electrolyte, the current density of the reaction decreased, and FE_CO_ significantly decreased (<50%, Figure S25, Supporting Information). Therefore, we believe that the atomically dispersed Zn centres play a leading role in the ECR process.

**Figure 4:**
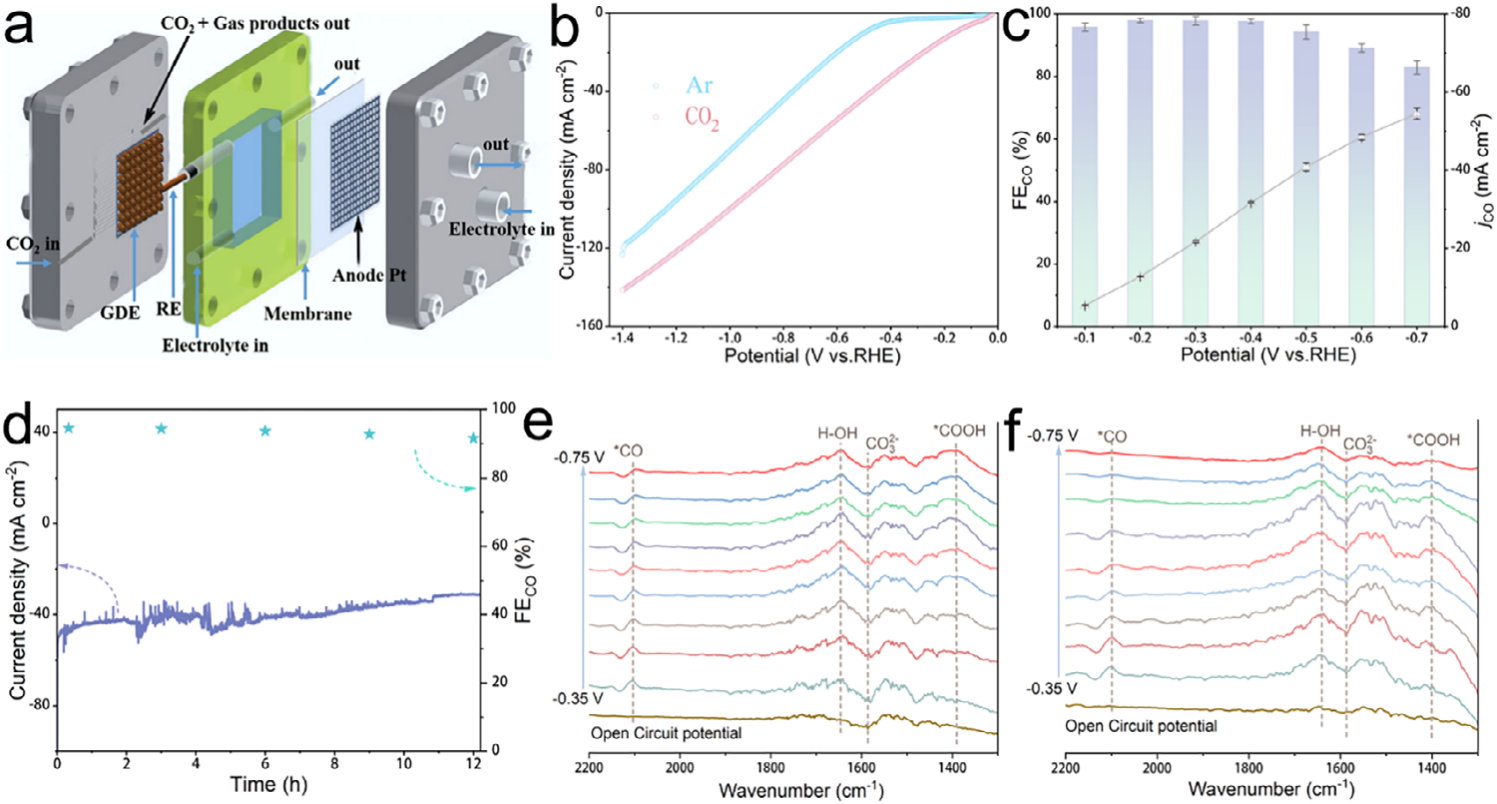
a) Flow cell structure diagram. b) LSV curve of ZnN_3_S_1_Cl/C catalyst in flow cell with 1 m KOH as electrolyte. c) FE_CO_ and *j*_CO_. d) Stability testing in a flow cell. e,f) ATR‐SEIRAS spectrum of (e) ZnN_4_/C and (f) ZnN_3_S_1_Cl/C.

To evaluate the practical applicability of the ZnN_3_S_1_Cl/C catalyst, its ECR performance was examined in a flow cell using 1 m KOH as the electrolyte (Figure S26, Supporting Information). As shown in **Figure** **4**a, the flow cell had a reduced distance between the cathode and anode, resulting in decreased reaction resistance. In addition, the direct supply of gaseous CO_2_ avoided the problem of the low solubility of CO_2_ in the liquid electrolyte, leading to a higher CO_2_ reduction current density (Figure 4b). Notably, in the flow cell, ZnN_3_S_1_Cl/C could maintain ≈100% FE_CO_ at −0.3 V (Figure 4c), and only CO and H_2_ were detected as the gas‐phase products (Figure S27, Supporting Information). Moreover, the ZnN_3_S_1_Cl/C catalyst could achieve an FE_CO_ of 94.01% and a *j*_CO_ of 45 mA cm^−2^ at −0.5 V. After continuous electrolysis at −0.5 V for 12 h, the FE_CO_ remained above 90% (Figure 4d).

To explore the ECR mechanism of the catalyst, we used attenuated total reflection surface‐enhanced infrared absorption spectroscopy (ATR‐SEIRAS) to comparatively evaluate the ECR performances of ZnN_4_/C and ZnN_3_S_1_Cl/C. In the ATR‐SEIRAS spectra, several characteristic vibrational bands were observed at ≈2106, 1641, 1583, and 1389 cm^−1^, which were assigned to the ^*^CO intermediate, H─OH bond, C═O bond (${\mathrm{CO}}_{3}^{2-}$), and ^*^COOH intermediate,^[^ ^25^ ^]^ respectively (Figure 4e and 4f). Generally, in the ECR for CO production, the formation of the ^*^COOH intermediate is the rate‐limiting step of the overall reaction. Notably, the generation of ^*^COOH was detected at a lower potential on ZnN_3_S_1_Cl/C, indicating that it has a lower onset potential than ZnN_4_/C. In addition, the generation of ^*^CO was detected on both the catalysts at −0.35 V. Differently, as the potential increased, the peak intensity corresponding to the ^*^CO formed on ZnN_4_/C remained unchanged, whereas that of the ^*^CO formed on ZnN_3_S_1_Cl/C gradually decreased, indicating that ^*^CO desorbed rapidly from the ZnN_3_S_1_Cl/C surface as the CO production rate increased in the reaction. Overall, the introduction of S and Cl atoms enhanced the adsorption of the ^*^COOH intermediate to the Zn centre in ZnN_3_S_1_Cl/C and the desorption of ^*^CO, thereby enhancing the ECR performance.

**Figure 5:**
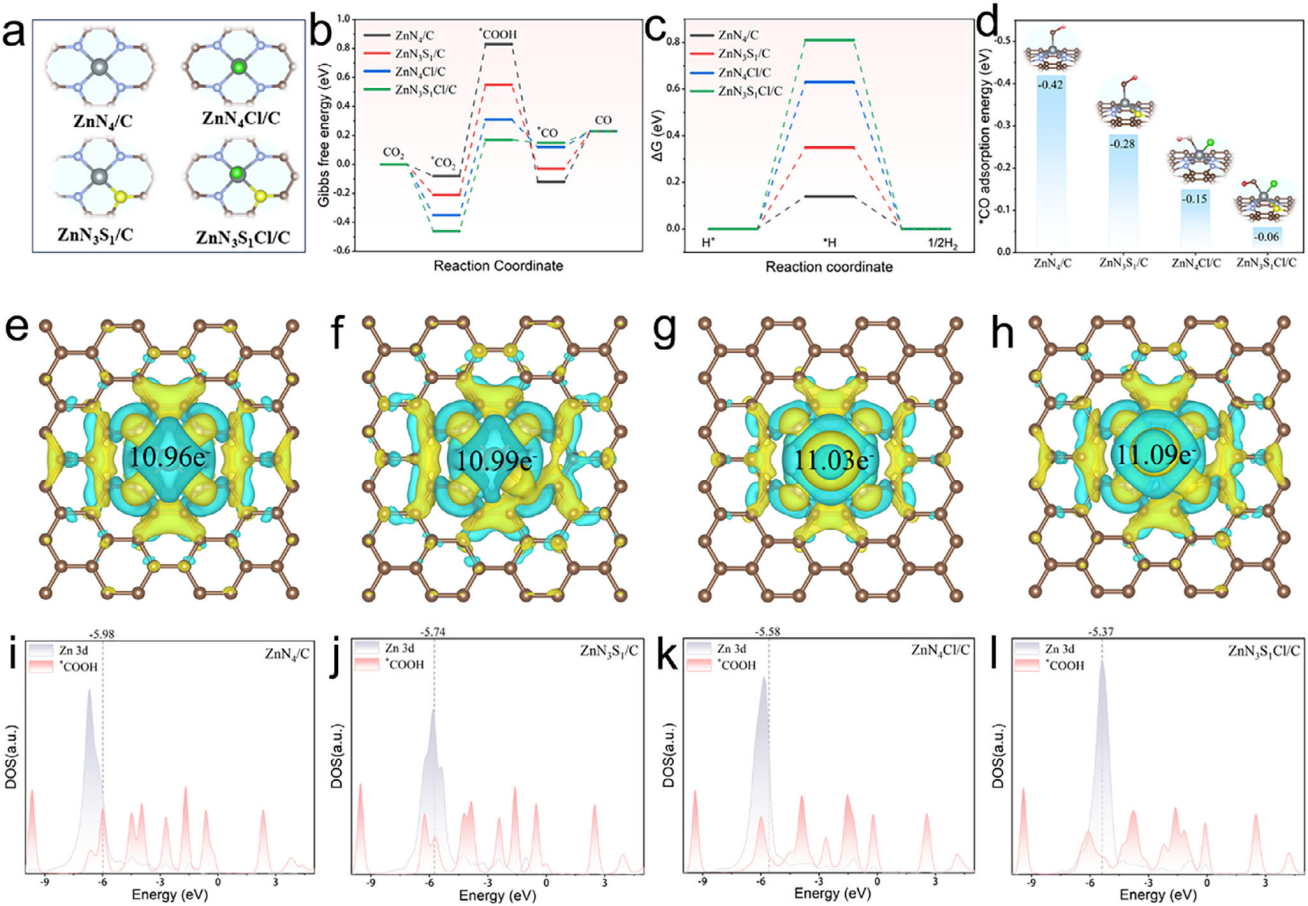
a) Optimized structural models of different catalysts. Calculated free energy diagrams for b) ECR and c) HER over different catalyst models. d) Desorption energy of ^*^CO. The charge density difference of e) ZnN_4_/C, f) ZnN_3_S_1_/C, g) ZnN_4_Cl/C, and h) ZnN_3_S_1_Cl/C models. Calculated DOS for (i) ZnN_4_/C, (j) ZnN_3_S_1_/C, (k) ZnN_4_Cl/C, and (l) ZnN_3_S_1_Cl/C with ^*^COOH adsorption.

To elucidate the regulatory mechanism of S and Cl atoms coordinated to single Zn atom sites in the ZnN_3_S_1_Cl/C catalyst and the influence of the symmetry breaking on the ECR performance, we further carried out density functional theory (DFT) calculations. The optimised structure of the ZnN_3_S_1_Cl/C catalyst (Figures S28–S30, Supporting Information) shows that the energy of the system is the lowest when Cl is in the axial position and S is in the asymmetric position. The most stable structure was selected for subsequent calculations (**Figure** **5**a). The ECR process for CO production involves multiple electron‐transfer steps. Generally, CO_2_ is captured and activated by the catalyst, and the formed ^*^CO_2_ undergoes an electron/proton transfer process to form the ^*^COOH intermediate. Subsequently, the ^*^COOH adsorbed on the active site is converted to ^*^CO through a similar process and desorbed from the catalyst (Figure S31, Supporting Information). As shown in Figure 5b, we calculated the Gibbs free energy change for each step. The formation of ^*^COOH from CO_2_ is the rate‐limiting step of the ECR to CO. The free energies required for this process on ZnN_3_S_1_Cl/C, ZnN_4_Cl/C, ZnN_3_S_1_/C, and ZnN_4_/C are 0.17, 0.31, 0.55, and 0.83 eV, respectively. The results indicate that the introduction of S and Cl is beneficial for reducing the formation energy of ^*^COOH, which is consistent with the results of the Tafel slope. In addition, the trend of the free energy change for H_2_ formation on various catalysts indicates that the change of the symmetric structure of Zn sites is conducive to inhibiting the HER (Figure 5c).

Furthermore, in the adsorption/desorption relationship of the CO_2_ reduction intermediates, a low ^*^CO desorption energy can prevent catalyst poisoning and is beneficial for the continuous progress of the reaction. Upon the introduction of a single heteroatom (S or Cl atom only), the ^*^CO desorption energy of ZnN_4_/C could be reduced from −0.42 to −0.28 eV for ZnN_3_S_1_/C and −0.15 eV for ZnN_4_Cl/C. When both S and Cl atoms were introduced simultaneously, the ^*^CO desorption energy of ZnN_3_S_1_Cl/C decreased to as low as −0.06 eV. These results demonstrate that the constructed axial and asymmetric structure of the ZnN_3_S_1_Cl/C catalyst enhances the ECR kinetics by promoting the formation of ^*^COOH and the desorption of ^*^CO, which is consistent with the ATR‐SEIRAS results.

The regulation mechanism of the S and Cl atoms on the ZnN_4_ symmetric sites was further investigated by calculating the charge density difference and projected density of states (DOS) of the different systems. The ZnN_4_ configuration exhibited a symmetric charge distribution in the density difference diagram (Figure 5e). When an S atom replaces an N atom of ZnN_4_ to form the ZnN_3_S_1_ asymmetric configuration, or a Cl atom exists in the axial position of ZnN_4_ to form ZnN_4_Cl, the charge density at the Zn site increases significantly owing to the destruction of the symmetric planar configuration of the system (Figure 5f,g). When both S and Cl atoms are introduced into ZnN_4_ to form a ZnN_3_S_1_Cl configuration with coupled axial and asymmetric coordination (Figure 5h), electrons are further redistributed, and the charge density of the system increases considerably from 10.96 e^−^ (ZnN_4_) to 11.09 e^−^ (ZnN_3_S_1_Cl). These changes lead to a reduction in the oxidation state of the Zn centre, which is consistent with the XPS and XAFS results. The interaction between the Zn site and reaction intermediates is altered by the electronic structure modulation induced by the introduced S and Cl atoms. Moreover, the intensity change between the metal centre and the intermediate was investigated using the DOSs of the Zn 3d and ^*^COOH 2p orbitals (Figure 5i–l). During the formation of the ^*^COOH intermediate, the introduction of both S and Cl can shift the Zn *d*‐band centre (*ε*_d_) toward the Fermi level (*E* = 0), increasing the overlap between the Zn (3d) and ^*^COOH (2p) orbitals. This enhances the adsorption strength of ^*^COOH on the Zn sites and reduces the energy barrier for ^*^COOH formation. Therefore, the coupling of the axial Cl coordination and asymmetric S coordination generates an electron‐rich Zn centre that stabilises ^*^COOH, thereby accelerating the conversion rate of CO_2_ to CO.

## 3 Conclusion

We successfully developed a new type of single‐atom ZnN_3_S_1_Cl/C catalyst with axial Cl and asymmetric S coordination. By breaking the traditional symmetric coordination configuration of the Zn atom in ZnN_4_ with the inclusion of Cl and S, the activity of the Zn centre was enhanced. The coordination structure of the Zn site in ZnN_3_S_1_Cl/C was determined by XPS and XAS, and the rationality of our catalyst design was verified through theoretical calculations. The charge density difference results indicated that the introduction of S and Cl atoms led to electron redistribution and an increase in the charge density around the Zn centre, resulting in a reduction in the valence state of Zn. This change in the electronic property of the Zn centre was consistent with the XPS and XAS results. Additionally, the introduction of both S and Cl can shift the Zn *d*‐band centre (*ε*_d_) toward the Fermi level (*E*_f_), increasing the overlap between the Zn (3d) and ^*^COOH (2p) orbitals, which enhances the adsorption strength of ^*^COOH on the Zn sites and reduces the energy barrier for ^*^COOH formation. The calculated ^*^CO desorption energy indicated that ZnN_3_S_1_Cl/C promotes the desorption of ^*^CO, which is beneficial for the continuous progress of the reaction. Consequently, ZnN_3_S_1_Cl/C achieved ≈100% FE_CO_ and long‐term stability over 240 h in an H‐cell, outperforming most of the previously reported SACs. Furthermore, in a flow cell, ZnN_3_S_1_Cl/C could achieve a maximum FE_CO_ of 99.02% at a low potential of −0.3 V. Thus, by introducing S and Cl heteroatoms, the coordination environment of a single‐atom metal centre was precisely adjusted, achieving an excellent ECR performance. This strategy may be applied to other metals (e.g., Ni, Fe, Co) commonly used in SACs for CO_2_ reduction. This work will provide key insights into the rational design and development of novel SACs for efficient and durable CO_2_ electroreduction.

## Conflict of Interest

The authors declare no conflict of interest.

## Supporting information



Supporting Information

## Data Availability

The data that support the findings of this study are available from the corresponding author upon reasonable request.
