# Supplementary material for: Axial and Asymmetric Coordination Coupling Adjust the Electronic Structure of Single‐Atom Zinc Sites for Efficient Electroreduction of Carbon Dioxide
Source: Adv Sci (Weinh). 2025 Sep 14;12(42):e09698. doi: 10.1002/advs.202509698 (PMC12622441; doi:10.1002/advs.202509698)
Supplement: Supplementary file 1 — Supporting Information [file ADVS-12-e09698-s001.docx]

**Supporting Information**

**Axial and Asymmetric Coordination Coupling Adjust the Electronic Structure of Single-Atom Zinc Sites for Efficient Electroreduction of Carbon Dioxide**

Cao Guo^a,b,1^, Feng Wang^a,1^, Abdukader Abdukayum^a^*, Qingde Chen^b^, Fengqin Chang^b^, Hongyi Li^c^*, Xuguang An^d^, Guangzhi Hu^a,b^* and Yujie Ma^e^*

^a^ Xinjiang Key Laboratory of Novel Functional Materials Chemistry, College of Chemistry and Environmental Sciences, Kashi University, Kashi 844000, China.

^b^ Qilu Lake Field Scientific Observation and Research Station for Plateau Shallow Lake in Yunnan Province, Institute for Ecological Research and Pollution Control of Plateau Lakes, School of Ecology and Environmental Science, Yunnan University, Kunming 650504, China.

^c^ State Key Laboratory of Chemistry and Utilization of Carbon Based Energy Resources, College of Chemistry, Xinjiang University, Urumqi, China.

^d^ School of Mechanical Engineering, Chengdu University, Chengdu 610106, China.

^e^ Department of Chemistry, University of Manchester, Manchester, M13 9PL, UK

^1^ These authors contributed equally.

*The corresponding author E-mail addresses: abdukadera@sina.com (A.A.), [lihongyi007007@163.com](mailto:lihongyi007007@163.com) (H.L.), [guangzhihu@ynu.edu.cn](mailto:guangzhihu@ynu.edu.cn) (G.H.) and [yujie.ma@manchester.ac.uk](mailto:yujie.ma@manchester.ac.uk) (Y.M.)

**Experimental Section**

**Materials**

2-methylimidazole, 2-mercapto-1-methylimidazole, Zn(CH_3_COO)_2_•2H_2_O, KHCO_3_, CH_3_OH and CH_3_CH_3_OH were purchased from Aladdin Co., Ltd. NH_3_·H_2_O was supplied by Tianjin Damao chemical reagent Co., Ltd. All chemicals were directly used without further purification.

**Catalyst synthesis**

Synthesis of ZIF-8. 1.73 g of Zn(CH_3_COO)_2_•2H_2_O was dissolved in 75 mL of methanol solution and recorded as solution A. 6.1 g of 2-methylimidazole was dissolved in 75 mL of methanol solution and recorded as solution B. The fully dissolved solutions A and B were mixed under stirring, and to improve the yield, they were heated and stirred in an oil bath at 60 ℃ for 24 h. Then, the product was collected by centrifugation, washed successively with methanol and ethanol, and dried overnight. Finally, a white product (ZIF-8) was obtained.

Synthesis of S@ZIF-8. 1.73 g of Zn(CH_3_COO)_2_•2H_2_O was dissolved in a 75 mL mixed solution of methanol and H_2_O (1:1), and recorded as solution C. 4.8 g of 2-methylimidazole and 1.3 g of 2-mercapto-1-methylimidazole were dissolved in 75 mL of methanol solution and recorded as solution D. The fully dissolved solutions C and D were mixed under stirring, and 2 mL of NH_3_·H_2_O was added to the mixture. Then, it was heated and stirred at 60 ℃ for 24 h. Finally, the product was collected by centrifugation, washed successively with methanol and ethanol, and dried overnight to obtain ~1.35g of the white product (S@ZIF-8).

Synthesis of ZnN_4_/C. ZIF-8 was heated to 1000 °C at 5 °C min^-1^ under a N_2_ atmosphere and held for 2 h.

Synthesis of ZnN_3_S_1_/C. S@ZIF-8 was heated to 1000 °C at 5 °C min^-1^ under a N_2_ atmosphere and held for 2 h.

Synthesis of ZnN_4_Cl/C. ZIF-8 and NaCl (mass ratio 1:1) were mixed and ground thoroughly, and then the resulting mixture was heated to 1000 ℃ at 5 ℃ min^-1^, under a N_2_ atmosphere and held for 2 h. Finally, the obtained black powder was washed with H_2_O and dried under vacuum at 60 °C for 12 h.

Synthesis of ZnN_3_S_1_Cl/C. 1.35 g of S@ZIF-8 was mixed with NaCl and milled; the mixture was then pyrolysed at 1000 ℃. The pyrolysed products were washed with water to remove the residual NaCl, obtaining black powder designated as ZnN_3_S_1_Cl/C (~ 0.7 g), with a yield of approximately ~51%. Potential challenges in scaling up could be the preparation of enlarged reactors.

**Catalyst characterization**

Scanning electron microscopy (SEM) images was performed on a Zeiss Sigma 300 microscope (Jena, Germany). Transmission electron microscopy (TEM) was performed on FEI Talos F200s. High–angle annular dark–field scanning transmission electron microscopy (HAADF–STEM) and energy–dispersive X–ray spectroscopy (EDS) elemental mapping were performed on FEI Themis Z. X–ray powder diffraction (XRD) patterns were detected on a Rigaku Ultima IV system using Cu Kα (λ = 1.54 nm) radiation in the 2θ range of 10°−90° at a scan rate of 5° min^−1^. The Fe content of catalysts was determined by Inductively Coupled Plasma-Mass Spectrometry (ICP–MS). X–ray photoelectron spectroscopy (XPS) was determined using a Thermo Fisher Scientific K–Alpha+ instrument with an Al Kα X–ray (1486.6 eV) radiation for excitation, and the binding energy was corrected by C1s value of 284.8 eV. The X–ray absorption spectroscopy (XAS) was obtained at the Institute of High Energy Physics Chinese Academy of Sciences.

**Computational details**

The density functional theory (DFT) calculations were carried out with the VASP code. The Perdew–Burke–Ernzerhof (PBE) functional within generalized gradient approximation (GGA) was used to process the exchange–correlation, while the projectoraugmented-wave pseudopotential (PAW) was applied with a kinetic energy cut-off of 500 eV, which was utilized to describe the expansion of the electronic eigenfunctions. The vacuum thickness was set to be 25 Å to minimize interlayer interactions. The Brillouin-zone integration was sampled by a Γ-centered 5 × 5 × 1 Monkhorst–Pack k-point. All atomic positions were fully relaxed until energy and force reached a tolerance of 1 × 10^-5^ eV and 0.03 eV/Å, respectively. The DFT-optimized Zn–N bond lengths are within 0.1 Å of the experimentally determined values (1.94–1.98 Å), demonstrating excellent agreement and indicating the adequacy of our model size and functional choice for electronic structure calculations. The dispersion corrected DFT-D method was employed to consider the long-range interactions [4]. Employing the climbing image nudged elastic band method (CI-NEB), we computed the minimum energy pathway of the cyclization reaction along with its corresponding activation barrier.

The adsorption energy (E_ads_) of a complex formed between two molecules, A and B, can be calculated using the following equation:

E_ads_ = E_complex_ - (E_A_ + E_B_)

Where: E_complex_ is the total energy of the molecular complex of A and B. E_A_ and E_B_ are the total energies of isolated molecules A and B, respectively.

The Gibbs free energy change (ΔG) was calculated by computational hydrogen electrode (CHE) model as follows:

ΔG = ΔE + ΔZPE − TΔS

where ΔE is the reaction energy obtained by the total energy difference between the reactant and product molecules absorbed on the catalyst surface and ΔS is the change in entropy for each reaction, ΔZPE is the zero-point energy correction to the Gibbs free energy. T represents room temperature (298.15 K).

**Electrochemical test**

Preparation of work electrode. The electrocatalyst ink was formulated by combining 10 mg of the catalyst with 1 mL of a dispersion comprised of 950 µL anhydrous ethanol and 50 µL of 5 wt.% Nafion solution, followed by ultrasonication for 30 minutes to ensure homogeneity. The working electrode was fabricated by depositing approximately 100 µL of this catalyst ink onto a carbon paper substrate measuring 1.0 × 1.0 cm^2^ active area.

To ensure the accuracy of the experiment, high–purity CO_2_ (99.999%) gas was injected for 30 min before each test to eliminate impurities from the electrolytic cell and achieve CO2 saturation in the electrolyte. A Metrohm Autolab M204 electrochemical workstation was used to collect the electrochemical signals. Cyclic voltammetry tests (100 mV s1 scan rate, 15 cycles) were performed to activate the catalyst and achieve a steady–state electrochemical reaction. The linear sweep voltammetry curve was then derived from the measurements at a scan rate of 100 mV s1 . In each experiment, the data were standardized to the Ag/AgCl reference electrode and reversible hydrogen electrode (RHE) using the following equation: ERHE = EAg/AgCl + 0.059 pH + 0.197 V.

Gaseous products from electrochemical experiments were analyzed by gas chromatography (GC). The Faraday efficiency (FE) of the gaseous product (CO/H_2_) is determined using the specified equation:

$$FE\left( \% \right)=\frac{Q_{CO/H_{2}}}{Q_{T}}=\frac{znF}{It}*100\%$$

In this equation, z represents the number of electrons transferred required to generate CO and H_2_ (z=2), n represents the number of moles of the resulting product, F represents Faraday's constant (96485 C/mol), I represents the current (A), and t is the duration of the reaction (s).

The CO turnover frequency (TOF) is the number of molecules of the reactant converted at each active site in unit time (s). It is an important parameter to measure the activity of catalyst and reflects the inherent activity of catalyst. The TOF of CO products is calculated using the following formula:

$$TOF=\frac{{j_{CO}}/{nF}}{{m*w}/M}*3600$$

Where, *j*_CO_ represents the partial current density of CO (A/cm^2^), m represents the catalyst load on the working electrode (g), and w represents the metal content of the catalyst, which is determined by ICP-MS. M is the atomic mass of the metal (g/mol).

**
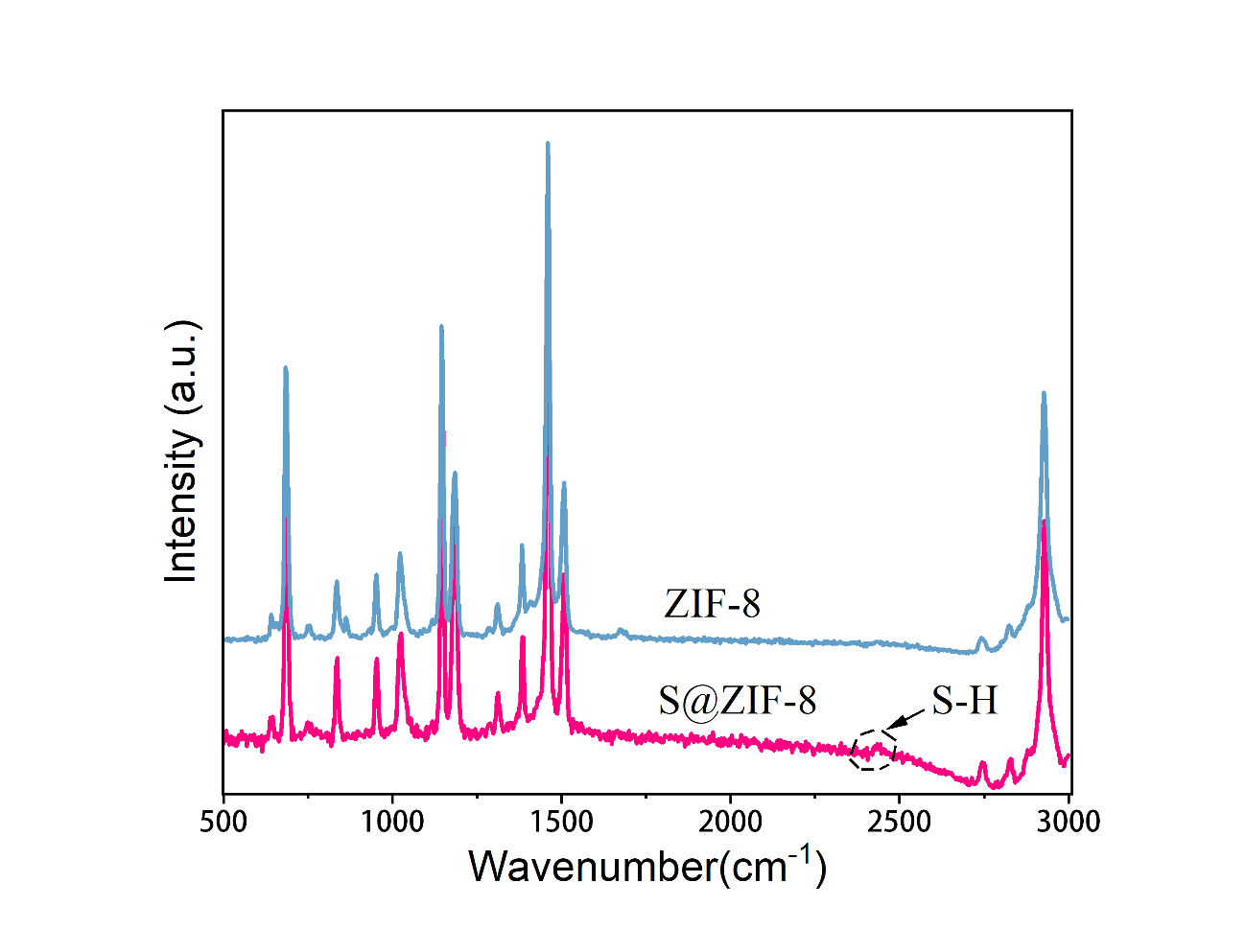
**

**Figure S1.** Raman spectra of ZIF-8 and S@ZIF-8.

**
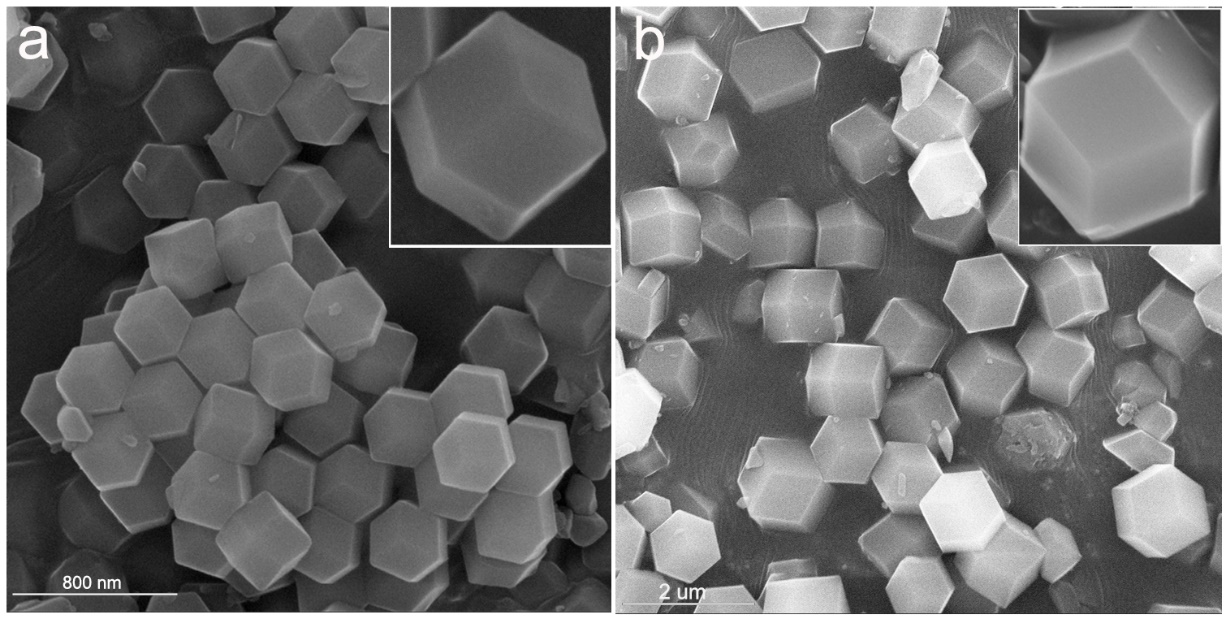
**

**Figure S2.** SEM images of (a) ZIF-8 and (b) S@ZIF-8.


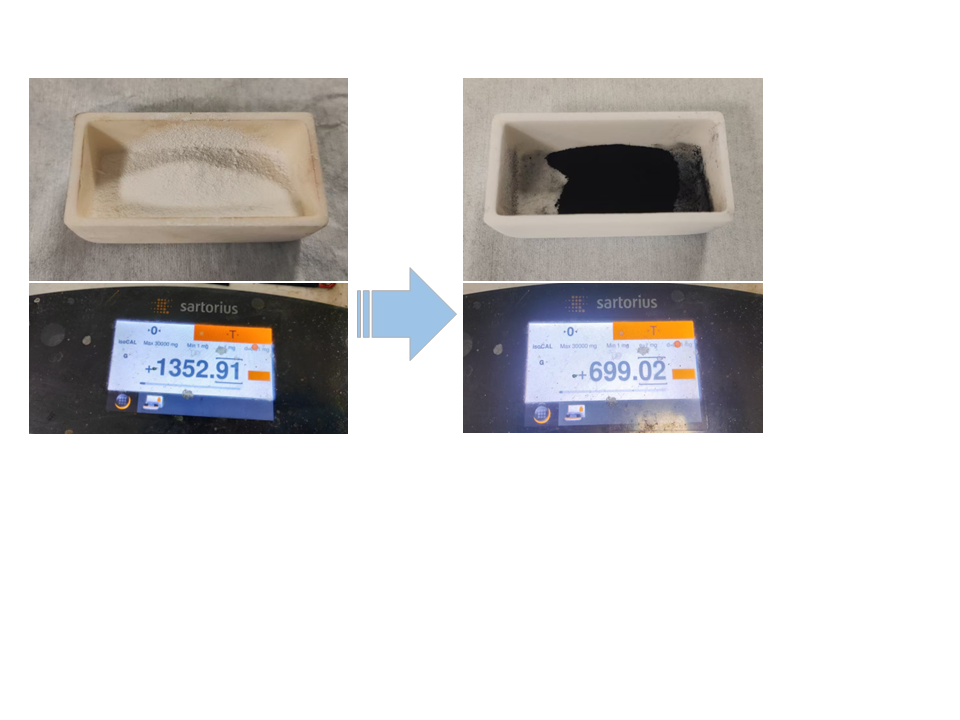


**Figure S3.** Images of the S@ZIF-8 and ZnN_3_S_1_Cl/C.

**
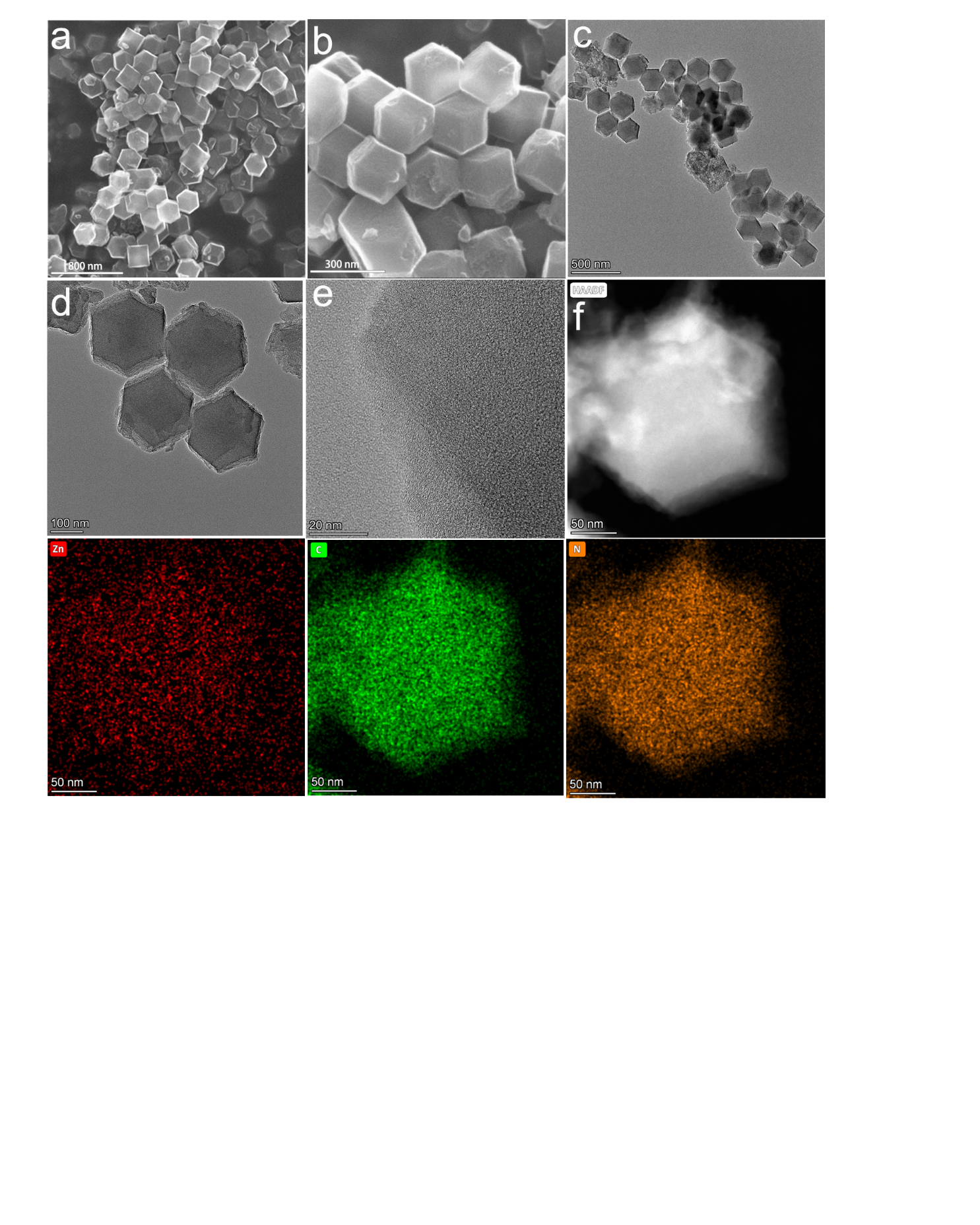
**

**Figure S4.** (a, b) SEM and (c-e) TEM images of ZnN_4_/C. (f) HAADF-STEM image and corresponding element mapping images of the ZnN_4_/C.


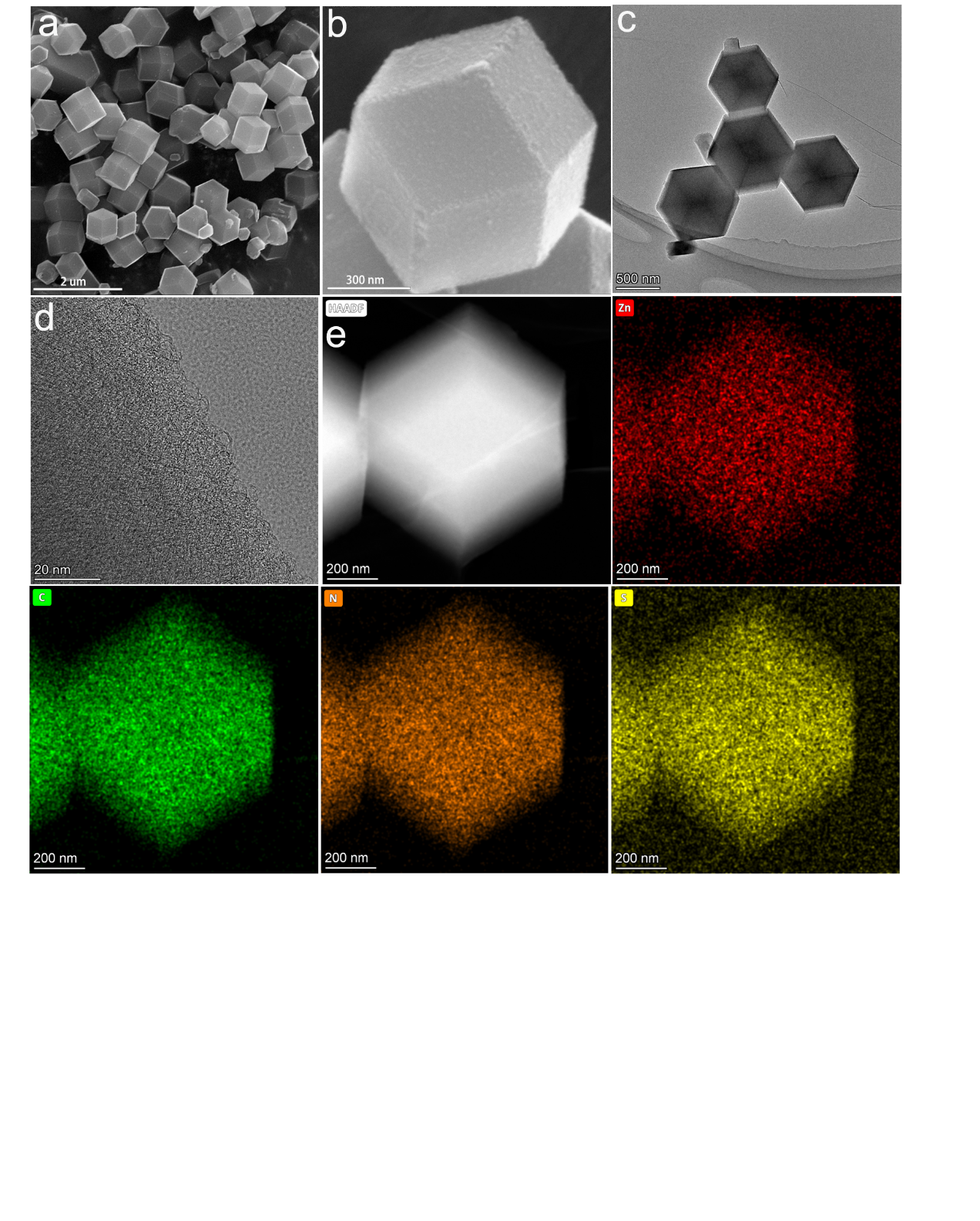


**Figure S5.** (a, b) SEM and (d, e) TEM images of ZnN_3_S_1_/C. (e) HAADF-STEM image and corresponding element mapping images of the ZnN_3_S_1_/C.


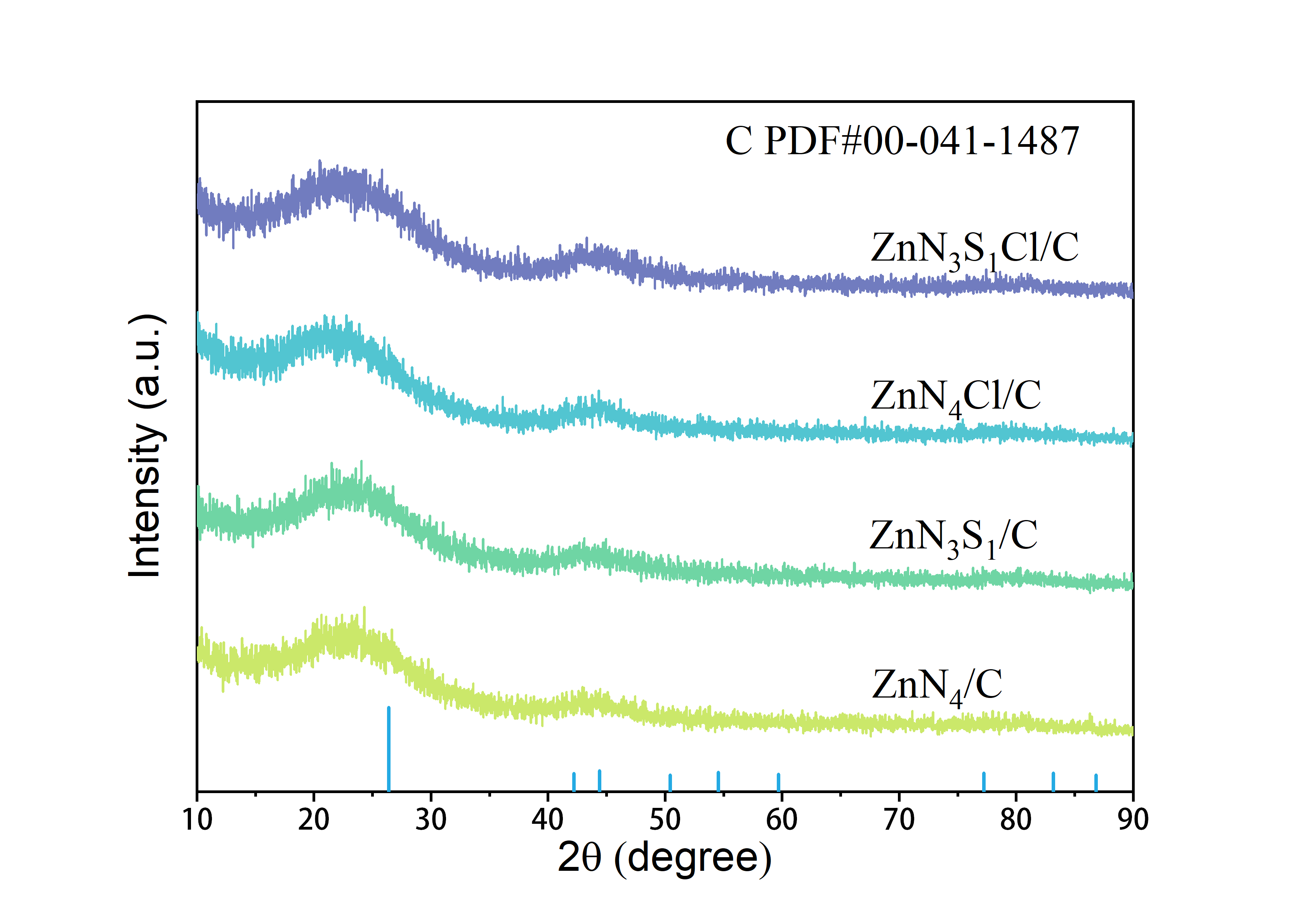


**Figure S6.** XRD patterns of the four catalysts.


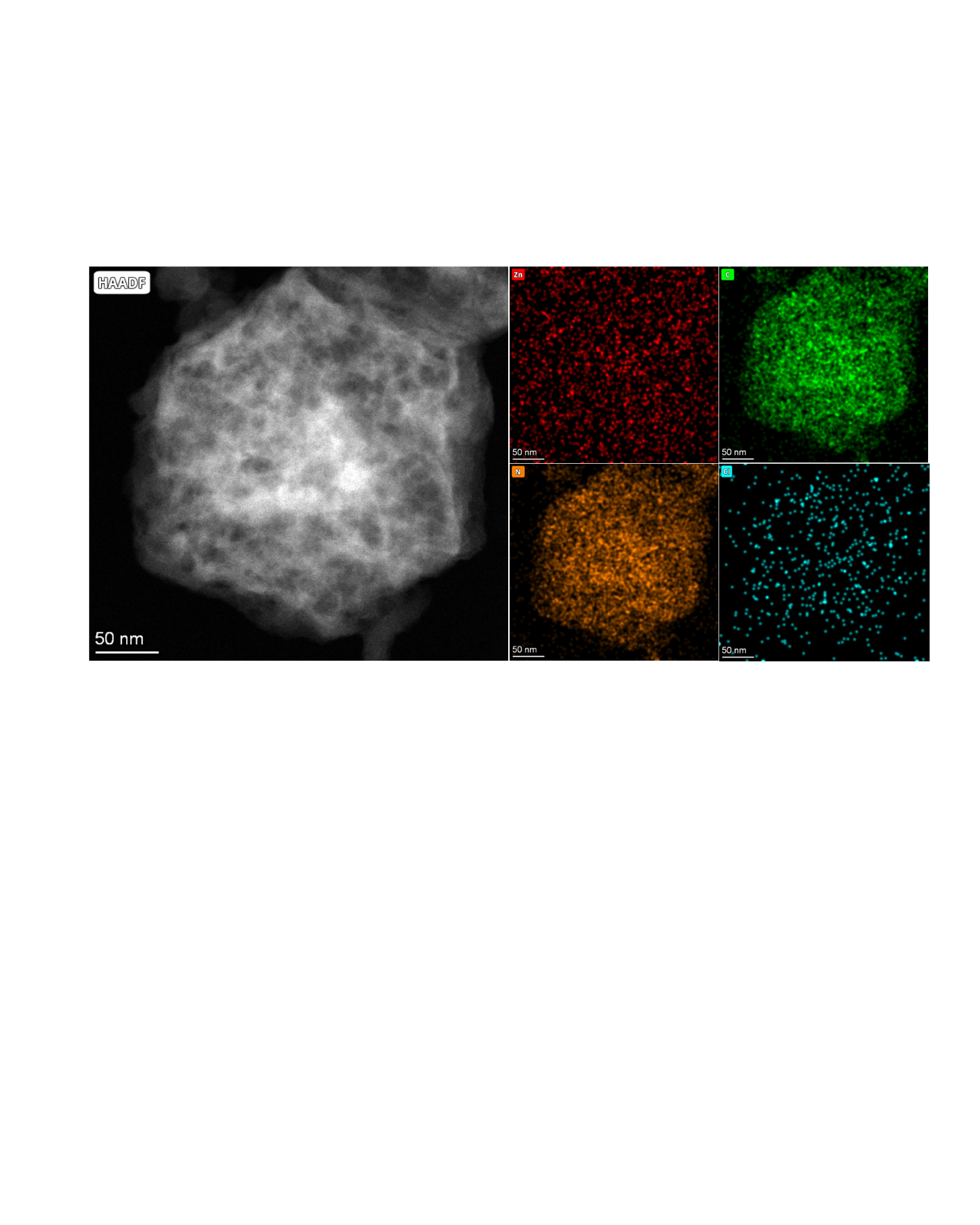


**Figure S7.** HAADF-STEM image and corresponding element mapping images of the ZnN_4_Cl/C.


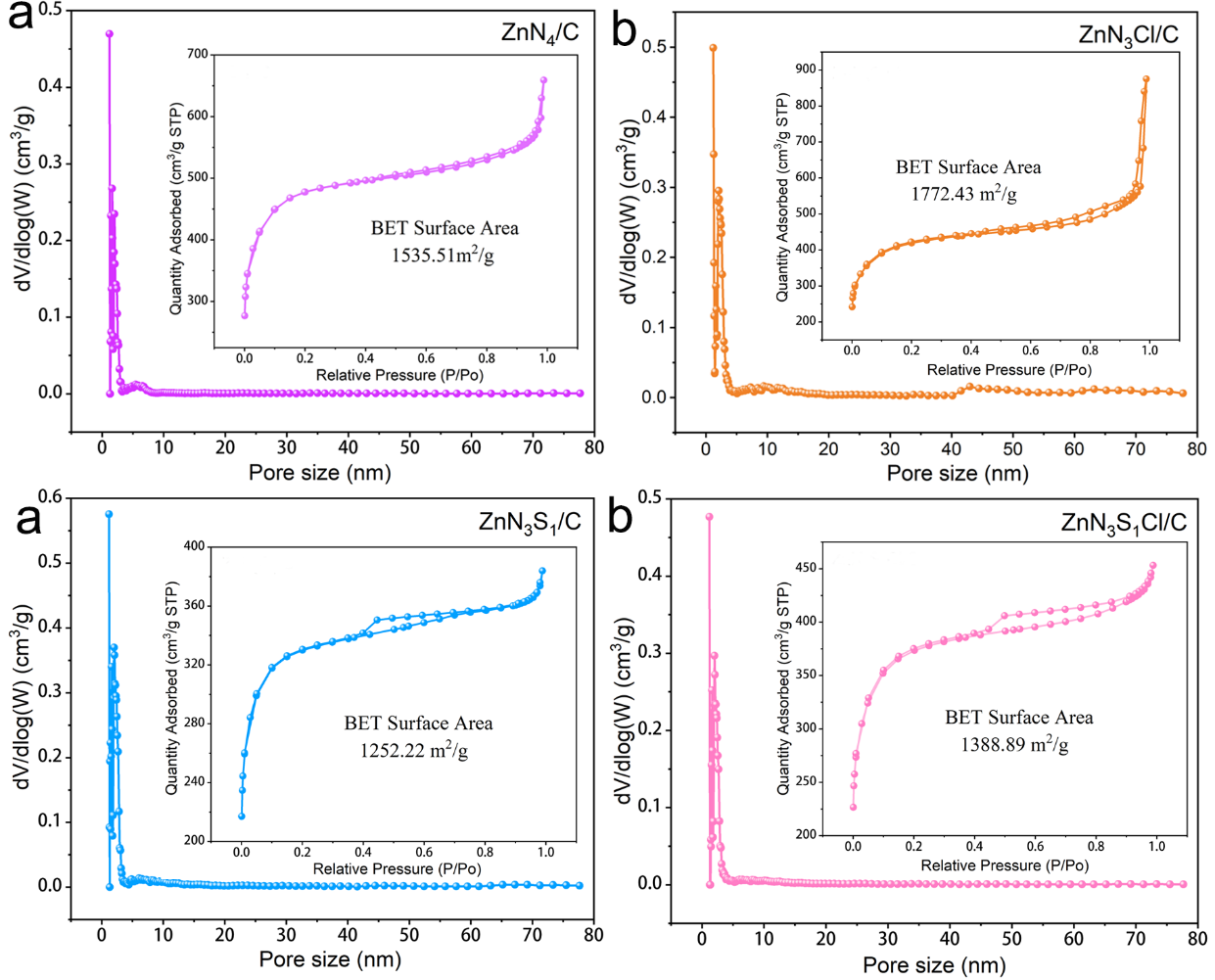


**Figure S8**. Pore size distribution (inset showing N_2_ adsorption/desorption isotherms) of (a) ZnN_3_S_1_/C and (b) ZnN_3_S_1_Cl/C.


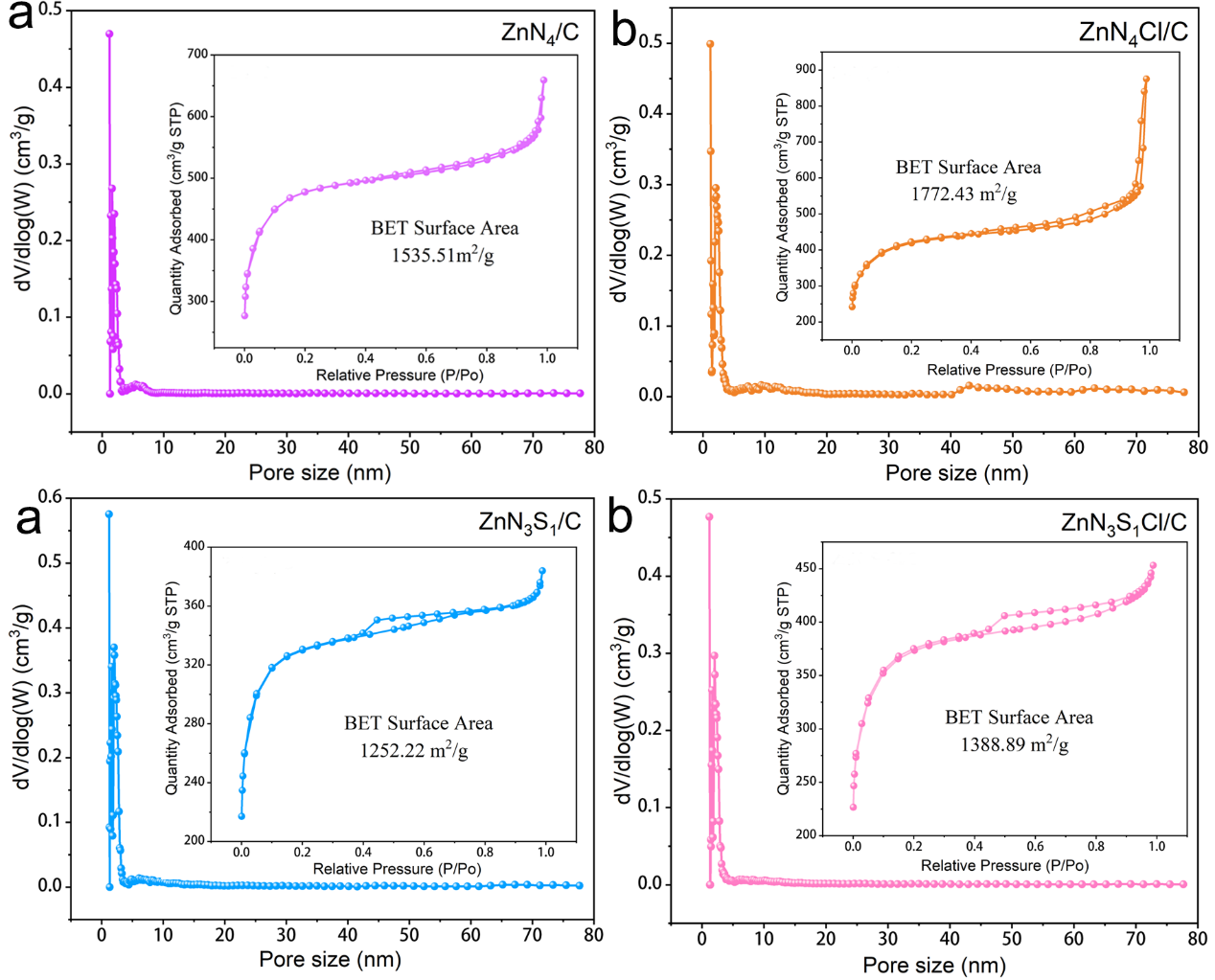


**Figure S9**. Pore size distribution (inset showing N_2_ adsorption/desorption isotherms) of (a) ZnN_4_/C and (b) ZnN_4_Cl/C.


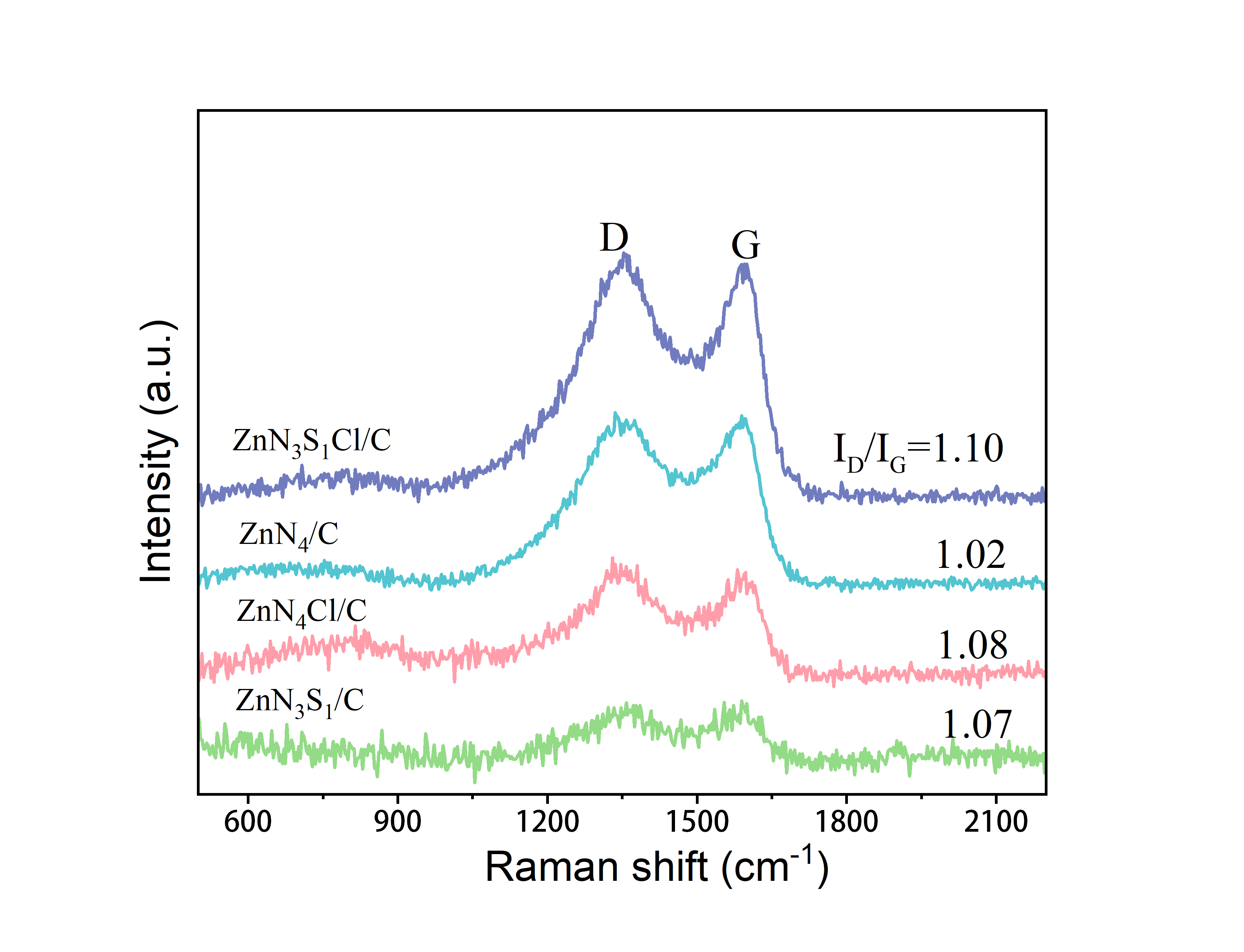


**Figure S10**. Raman spectra of four catalysts.


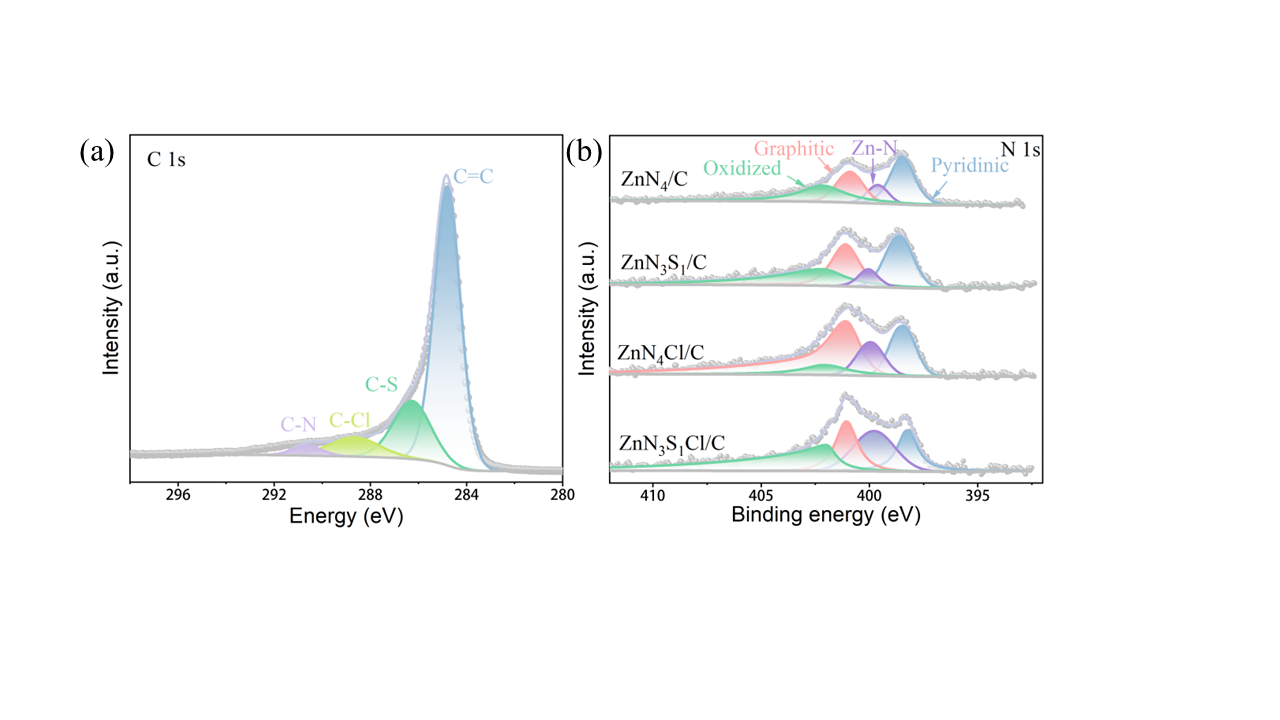


**Figure S11.** (a) C 1s XPS spectra of ZnN_3_S_1_Cl/C. (b) N 1s XPS spectra of ZnN_4_/C, ZnN_3_S_1_/C, ZnN_4_Cl/C and ZnN_3_S_1_Cl/C


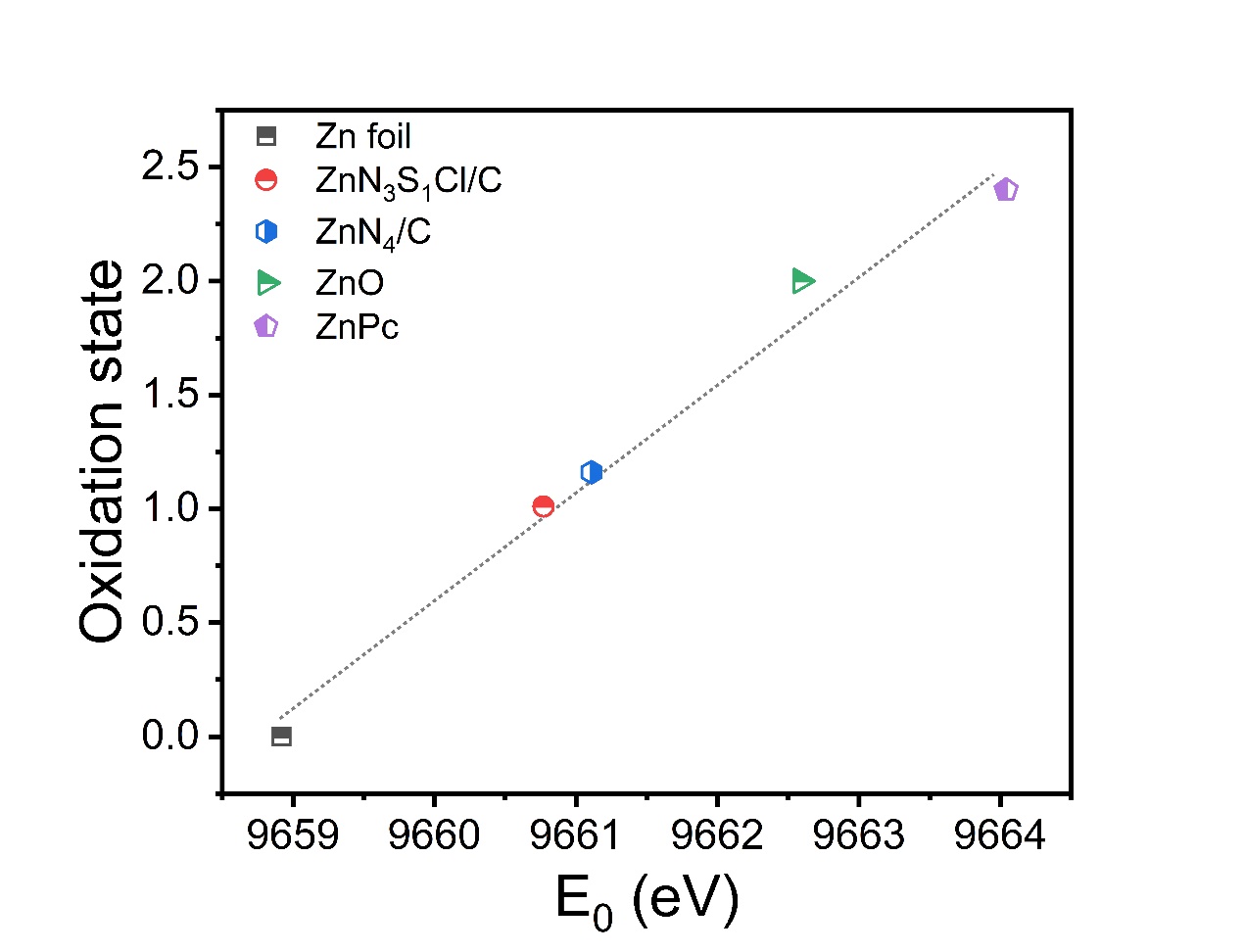


**Figure S12.** Liner fitting for Zn valences derived from corresponding Zn K-edge XANES spectra.


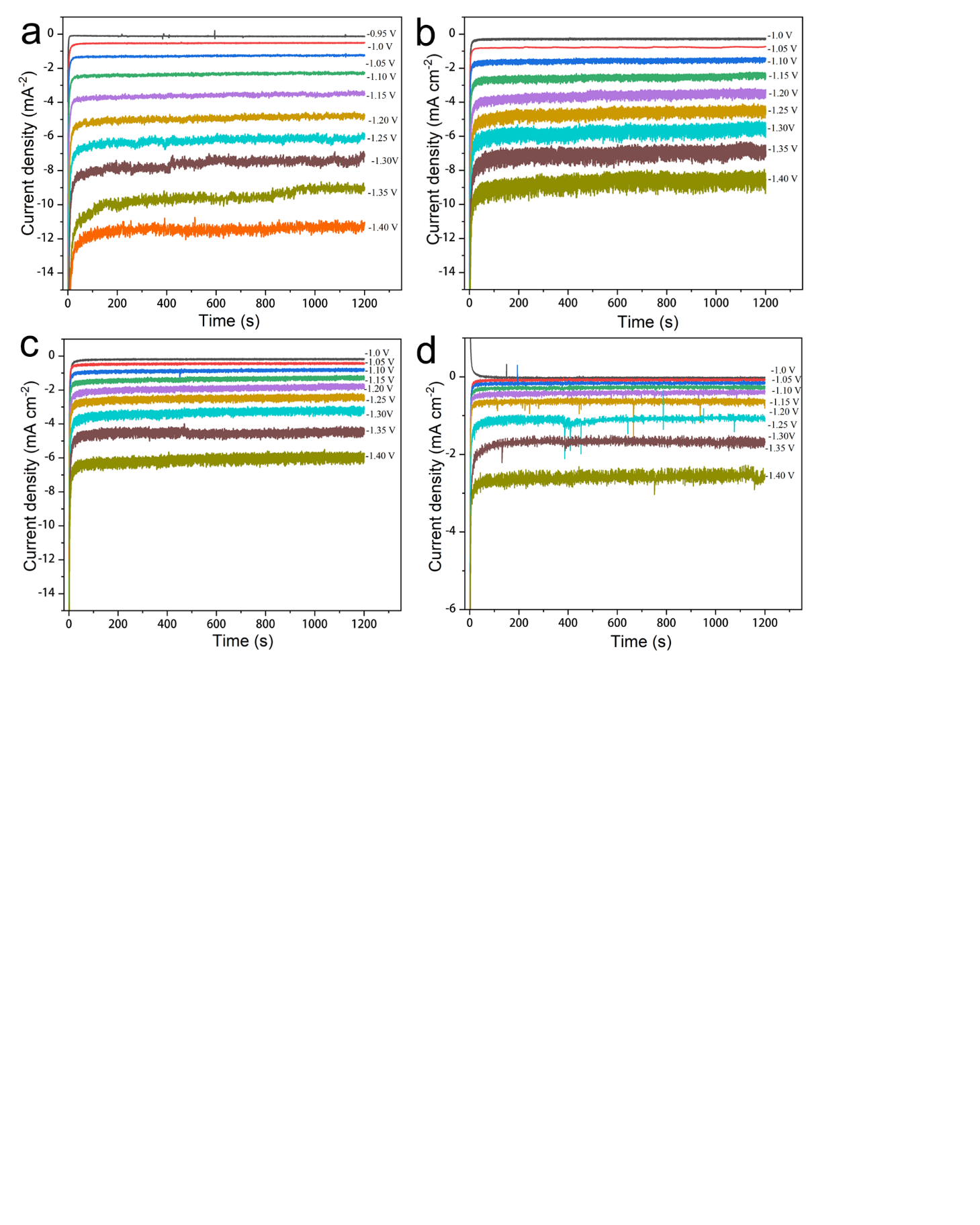


**Figure S13.** i-t curves of catalysts in CO_2_-saturated 0.5 M KHCO_3_ solution. (a) ZnN_3_S_1_Cl/C. (b) ZnN_4_Cl/C. (c) ZnN_3_S1/C. (d) ZnN_4_/C.


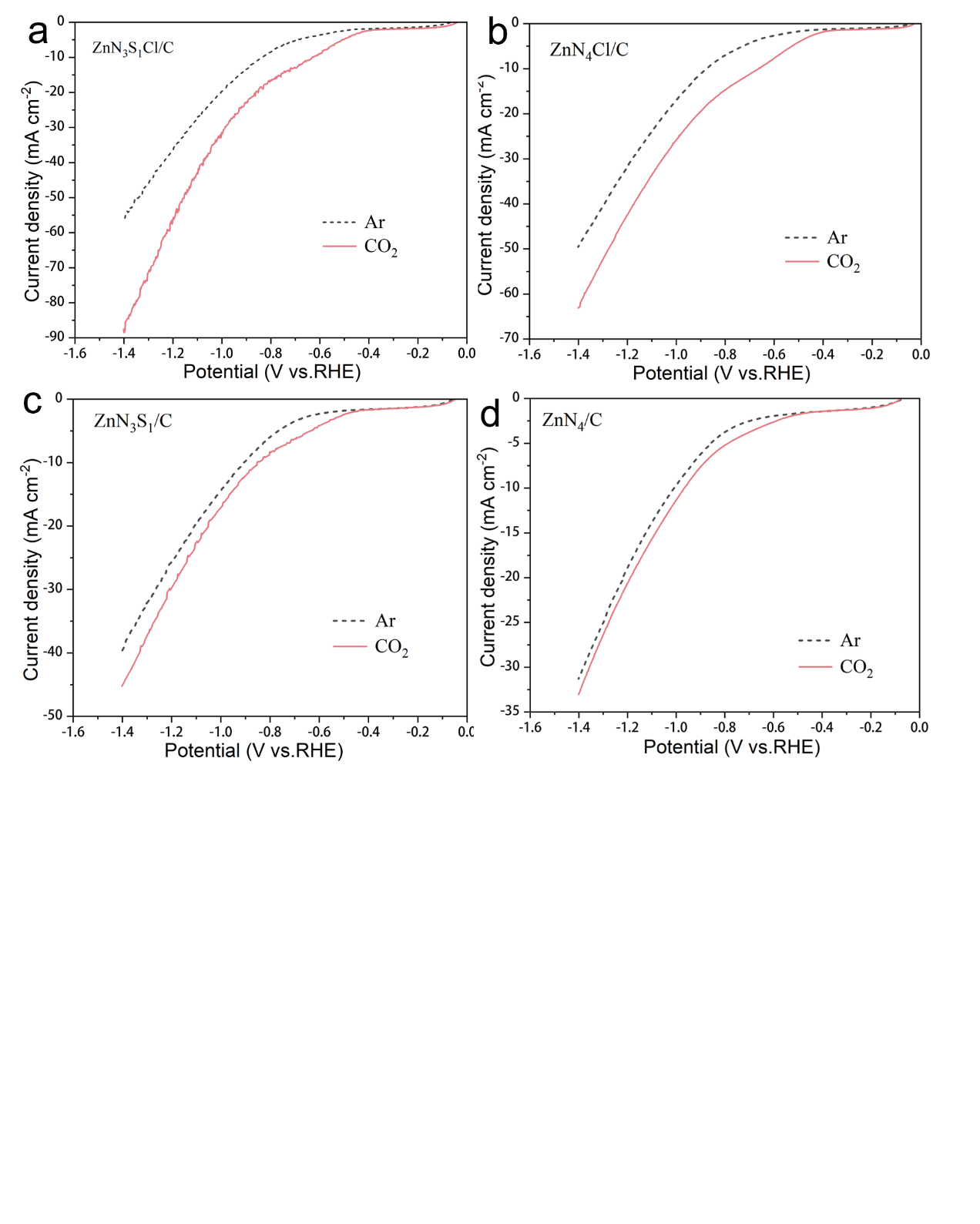


**Figure S14.** LSV curves of catalysts in H-cell. (a) ZnN_3_S_1_Cl/C. (b) ZnN_4_Cl/C. (c) ZnN_3_S1/C. (d) ZnN_4_/C.


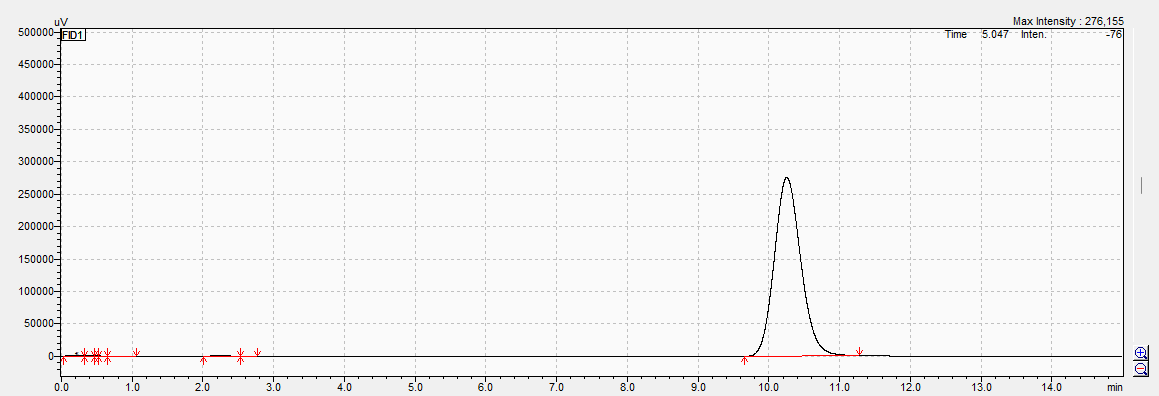


**Figure S15.** Schematic diagram of product peaks detected by GC at -0.55 V vs. RHE in H-Cell.


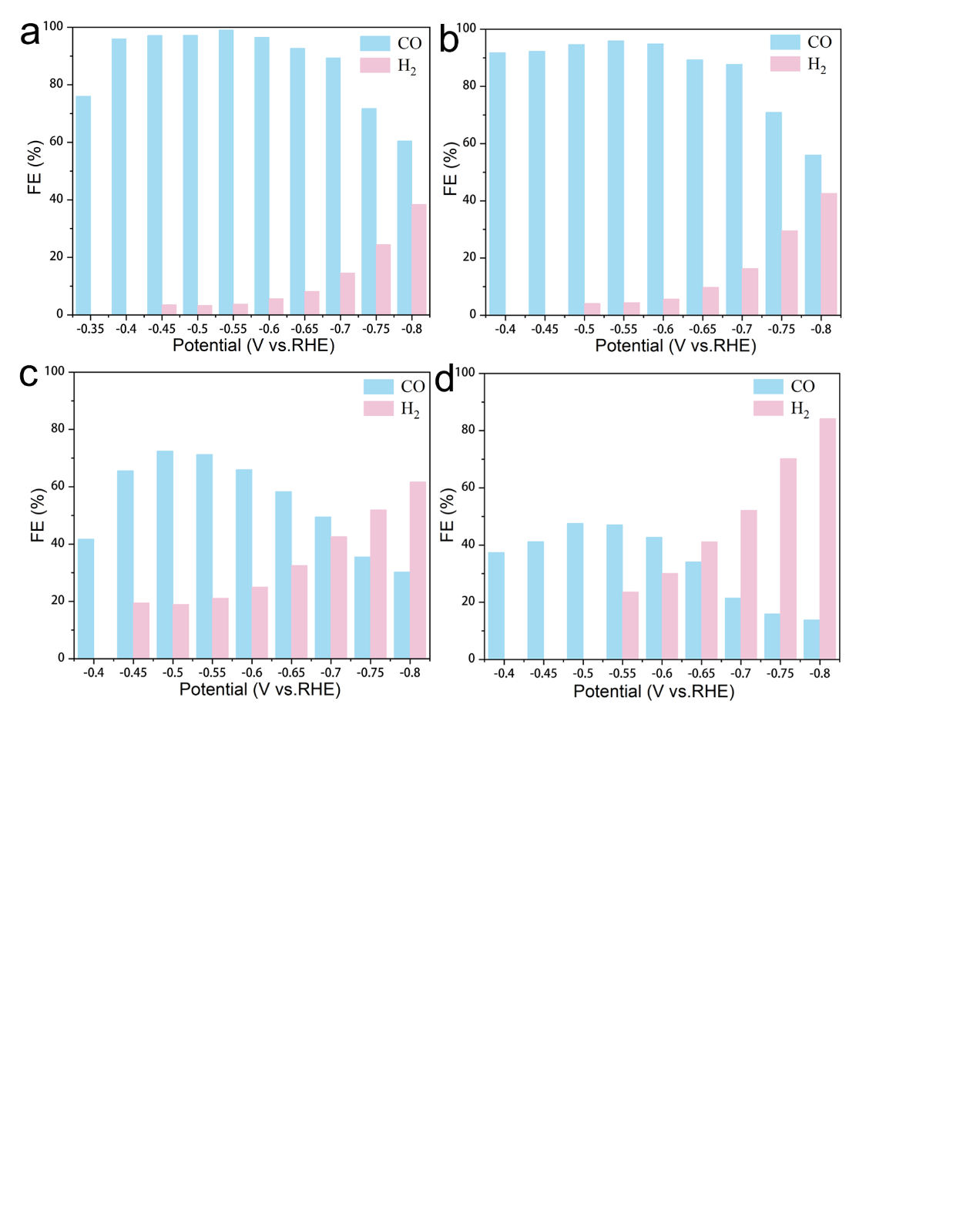


**Figure S16.** CO and H_2_ Faraday efficiencies. (a) ZnN_3_S_1_Cl/C. (b) ZnN_4_Cl/C. (c) ZnN_3_S_1_/C. (d) ZnN_4_/C.


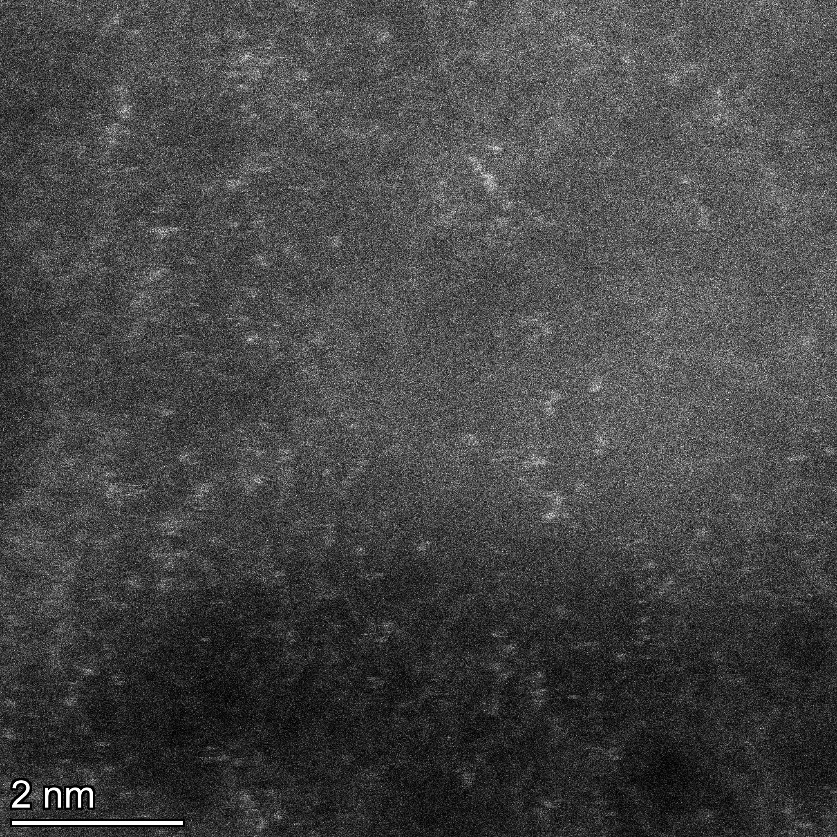


**Figure S17.** AC HAADF-STEM image of ZnN_3_S_1_Cl/C after a long stability test.


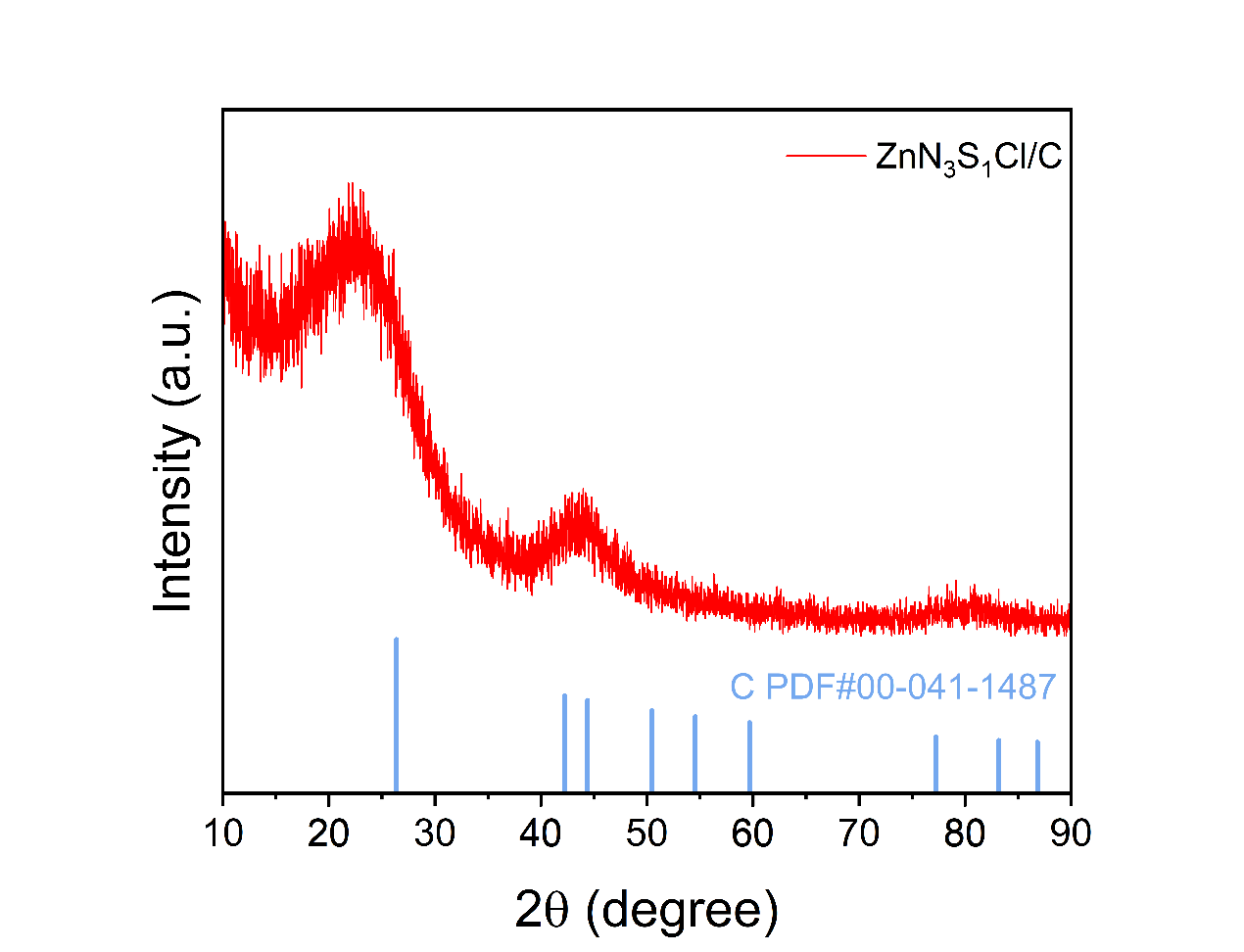


**Figure S18**. XRD patterns of ZnN_3_S_1_Cl/C after a long stability test.


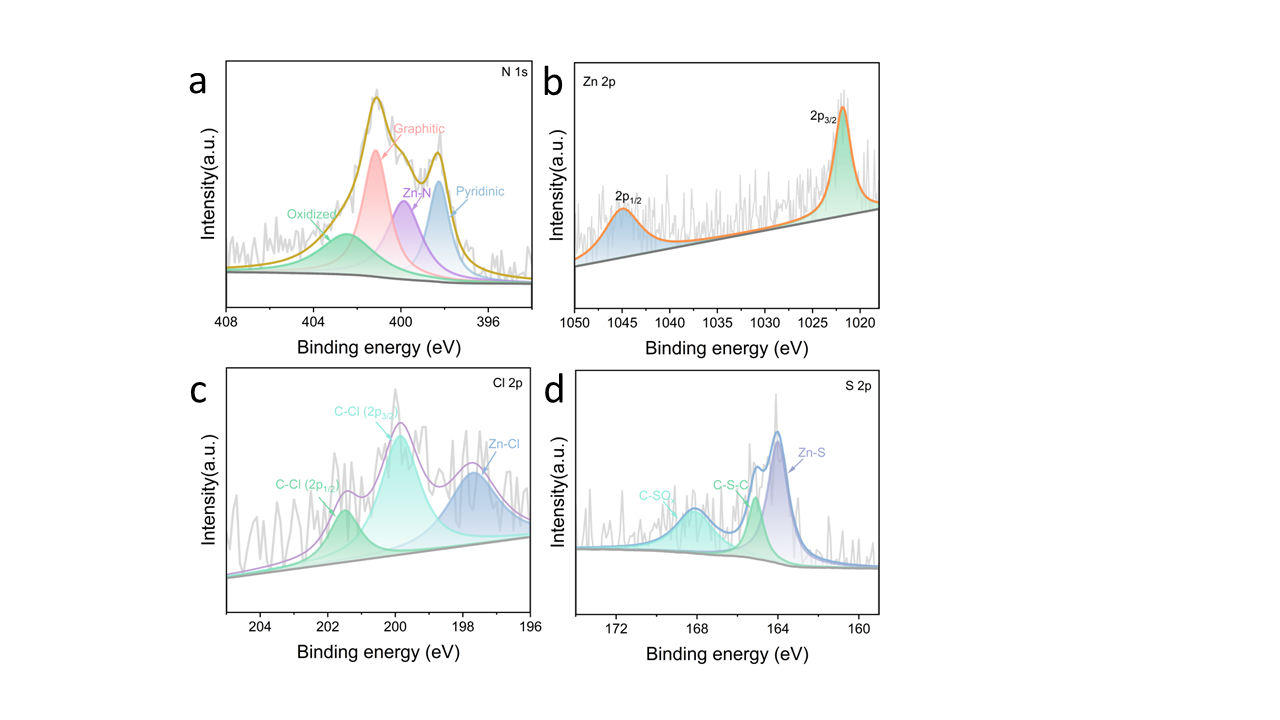


**Figure S19**. XPS spectra of ZnN_3_S_1_Cl/C after a long stability test.


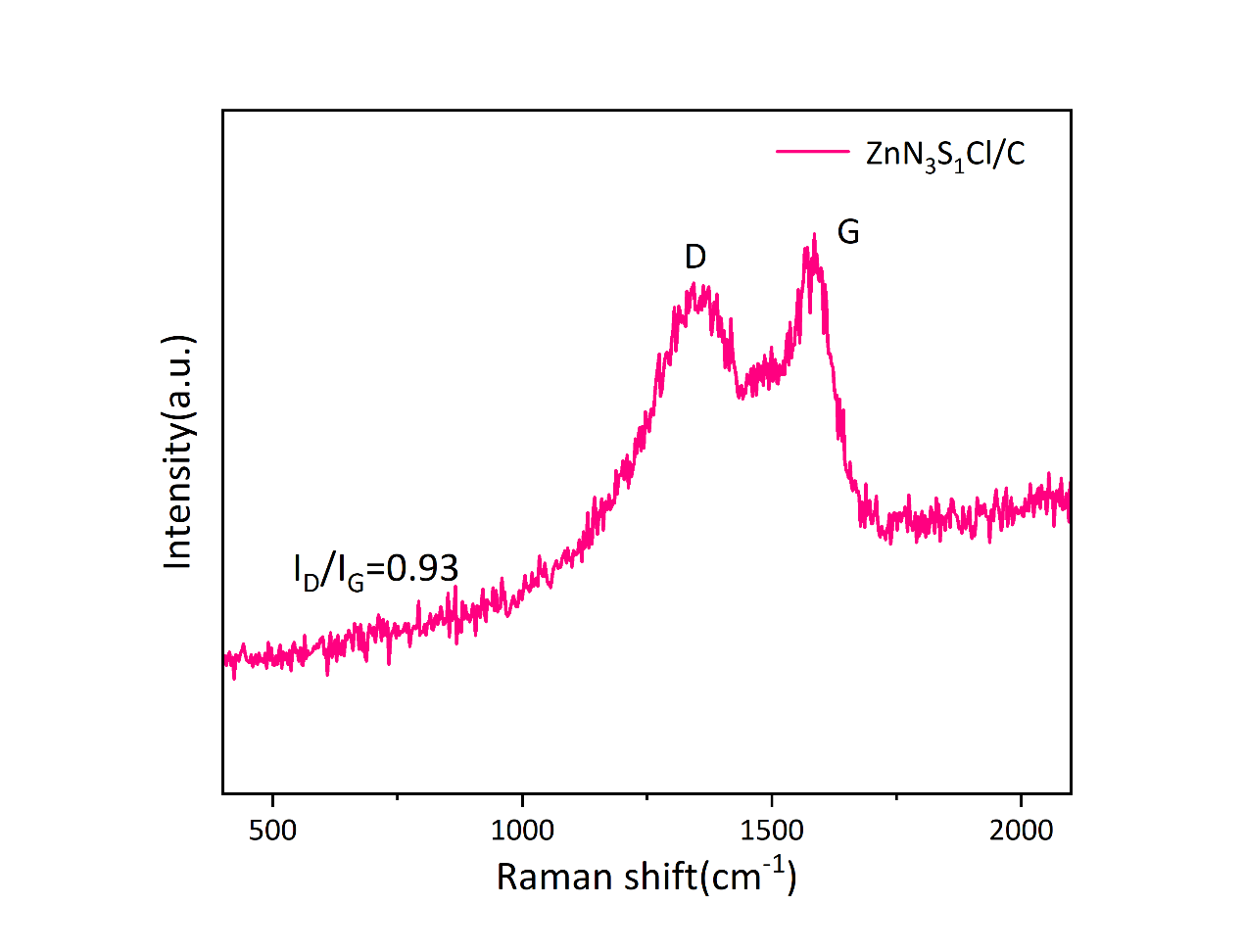


**Figure S20**. Raman data of ZnN_3_S_1_Cl/C after a long stability test.


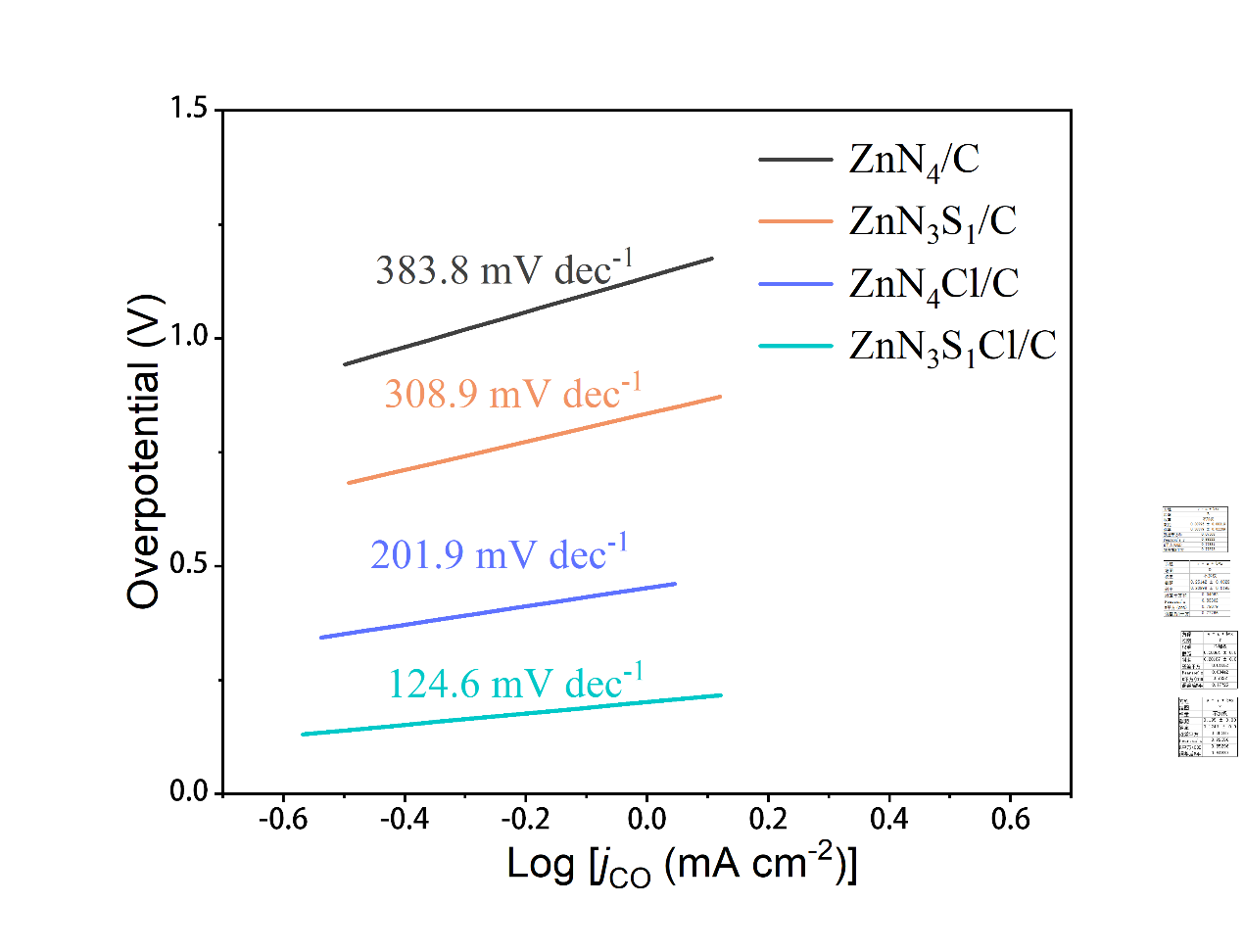


**Figure S21.** Tafel plots of catalysts.


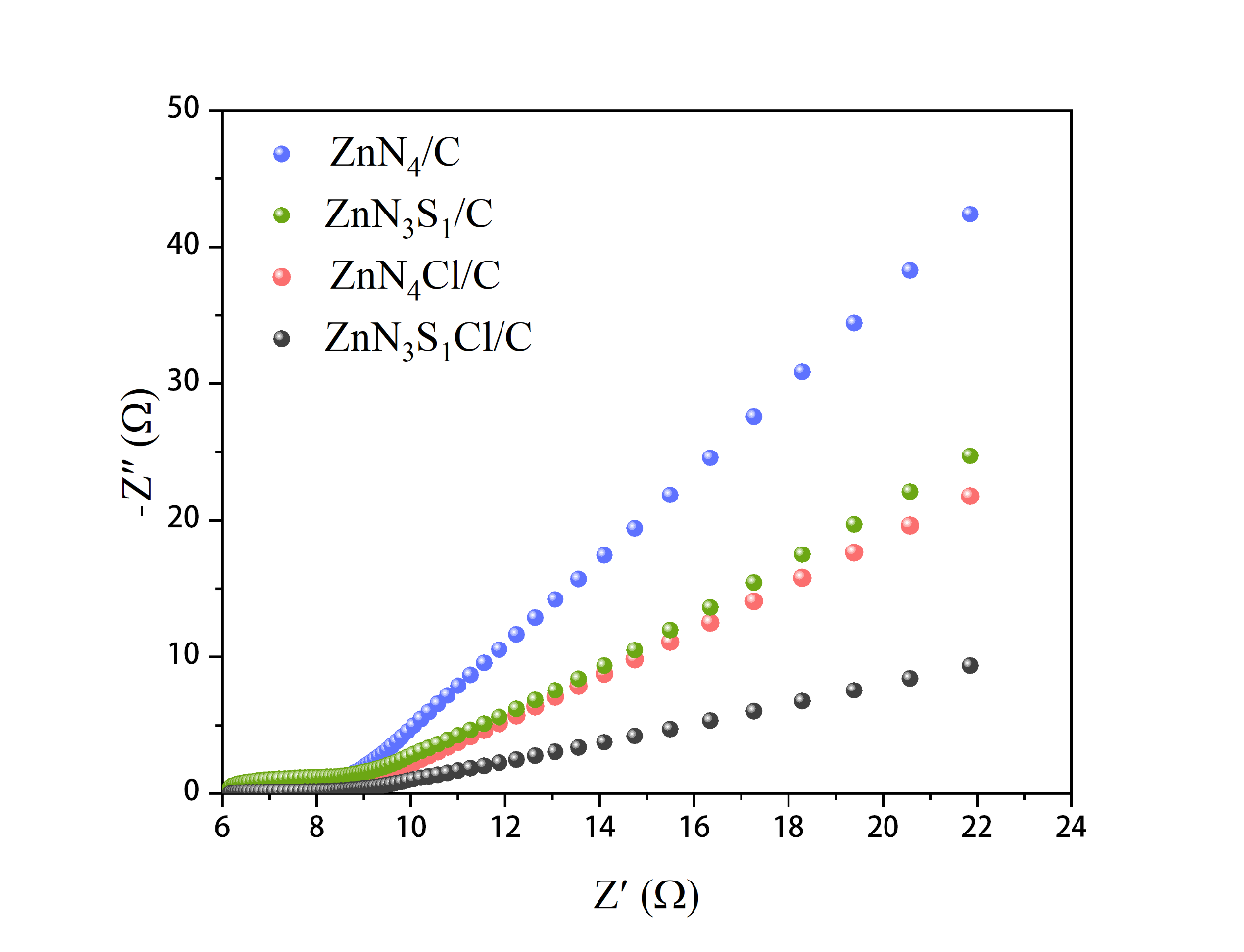


**Figure S22.** EIS of catalysts.


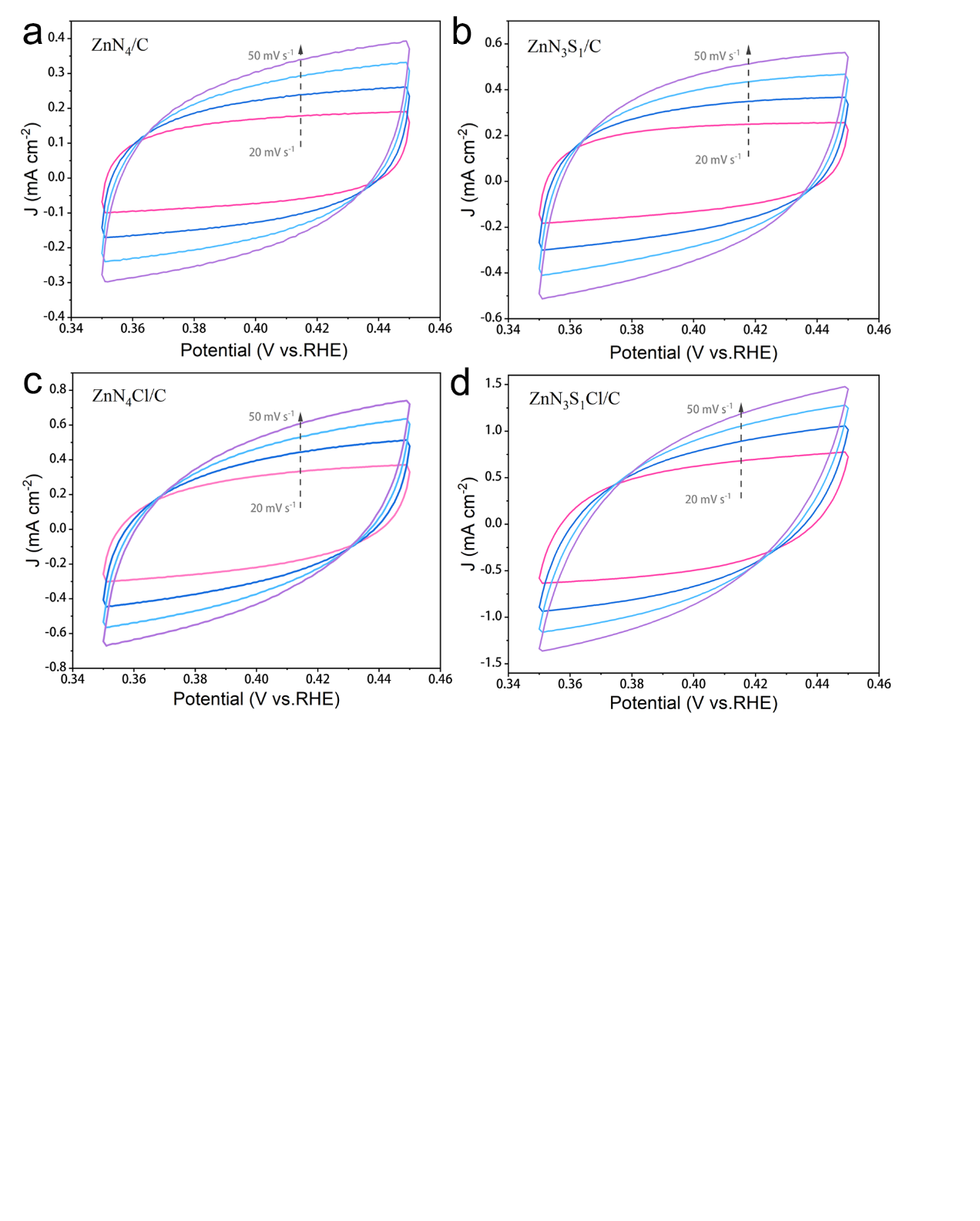


**Figure S23.** CV curves for (a) ZnN_4_/C, (b) ZnN_3_S_1_/C, (c) ZnN_4_Cl/C, and (d) ZnN_3_S_1_Cl/C with various scan rates for CO_2_ electroreduction.


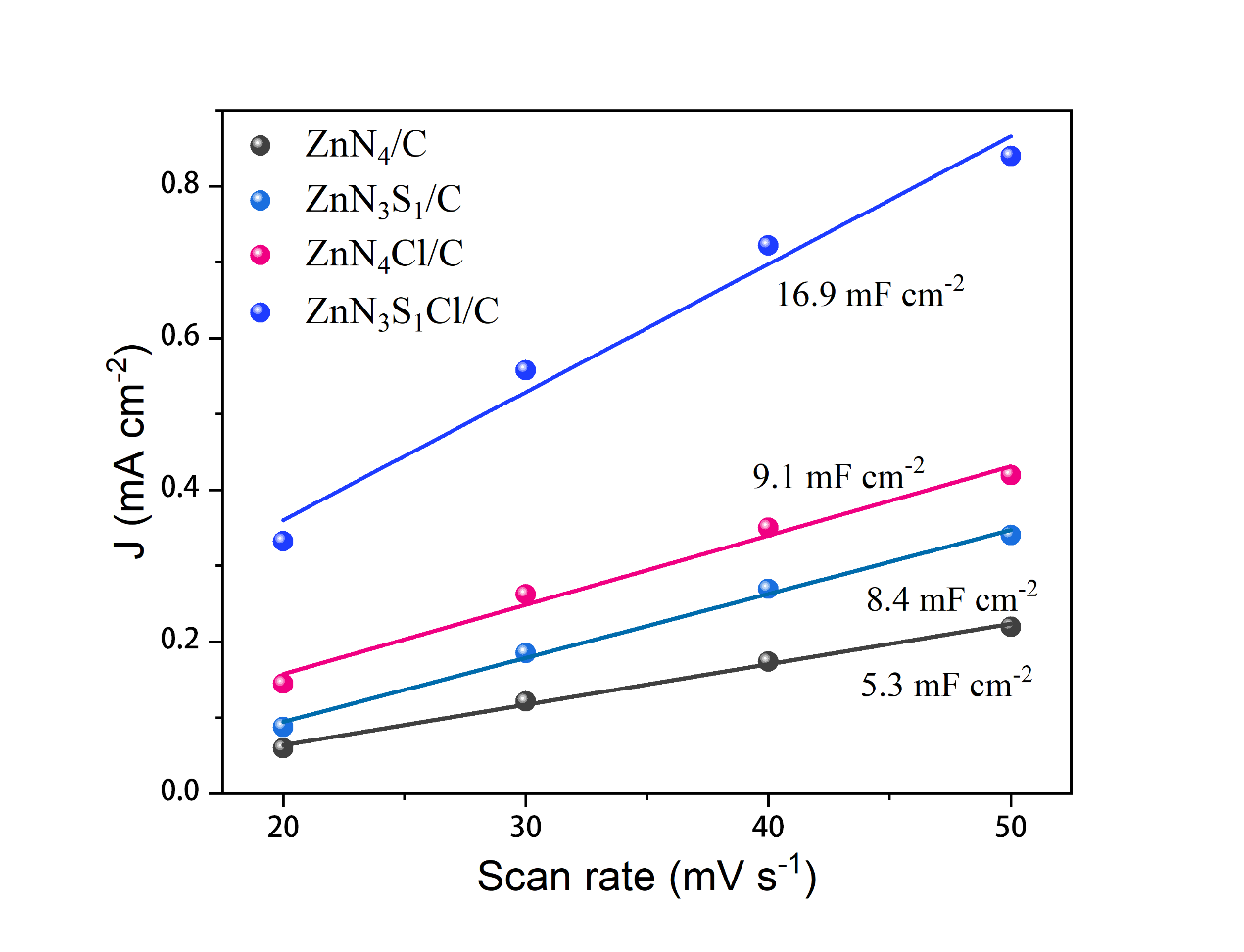


**Figure S24.** ECSAs of catalysts.


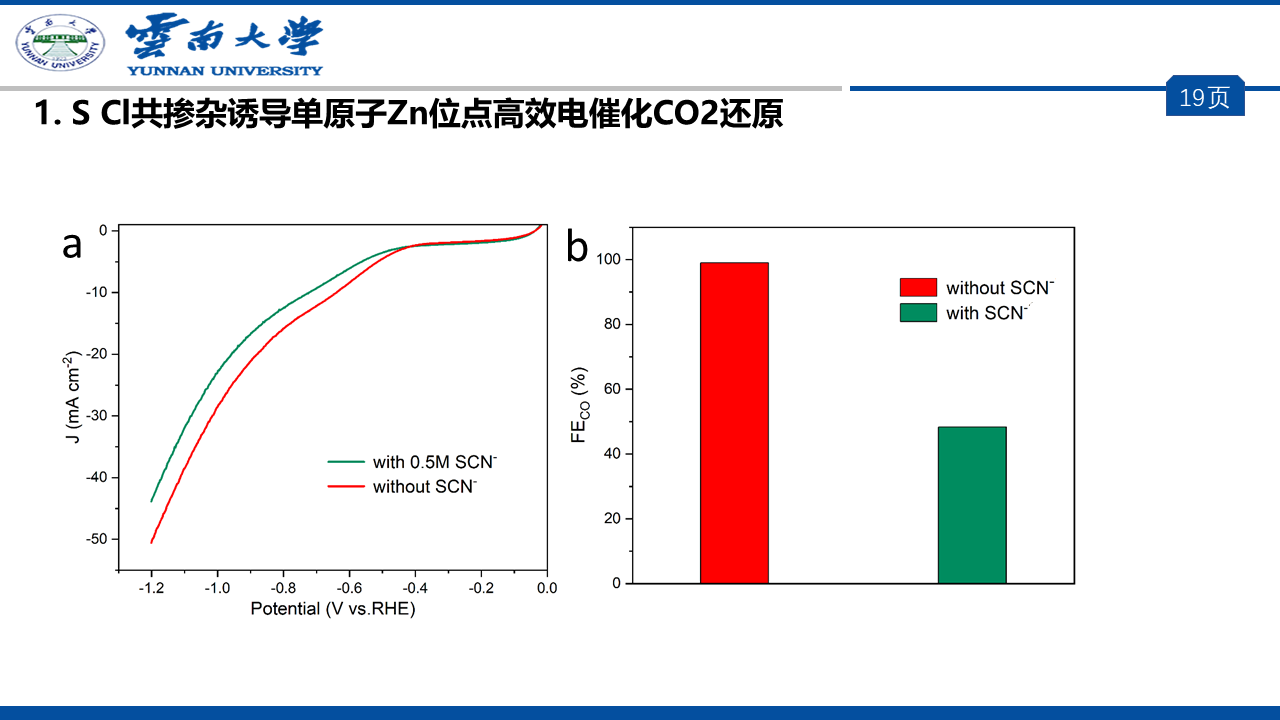


**Figure S25.** (a) LSV curves in 0.5 M KSCN solution, (b) FE_CO_ in 0.5 M KSCN solution.


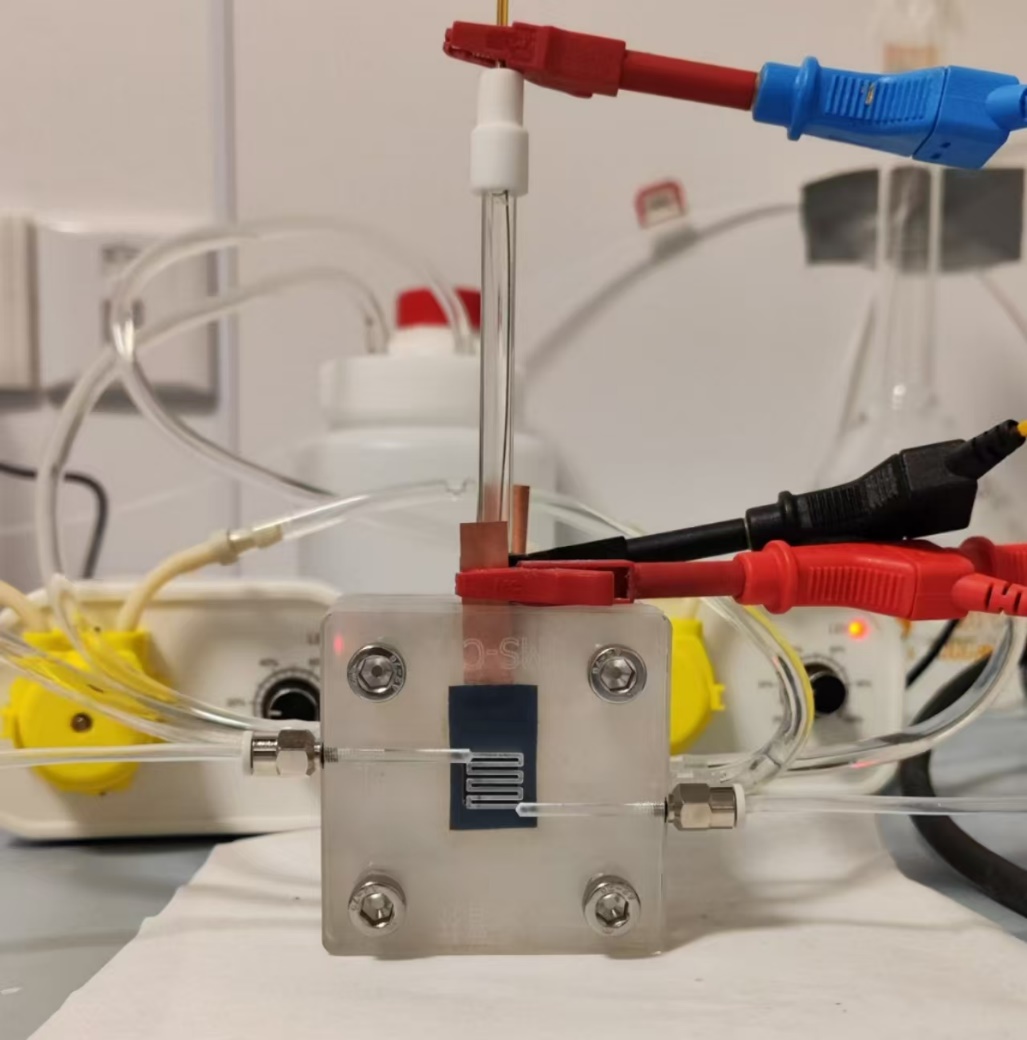


**Figure S26.** Digital images of flow cell used in this work.


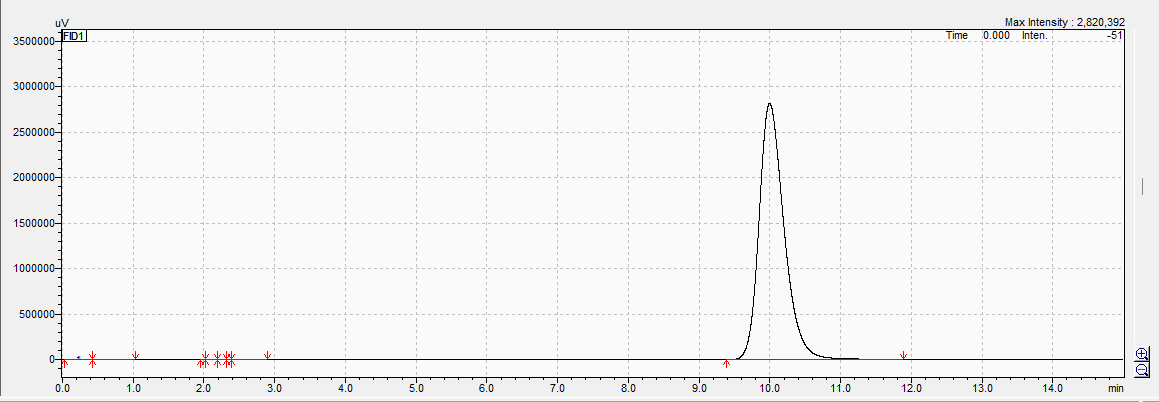


**Figure S27.** Schematic diagram of product peaks detected by GC at -0.55 V vs. RHE in flow cell.

.


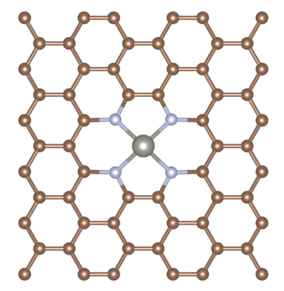


**Figure S28.** Optimized structural models of ZnN_4_/C (539.66eV).


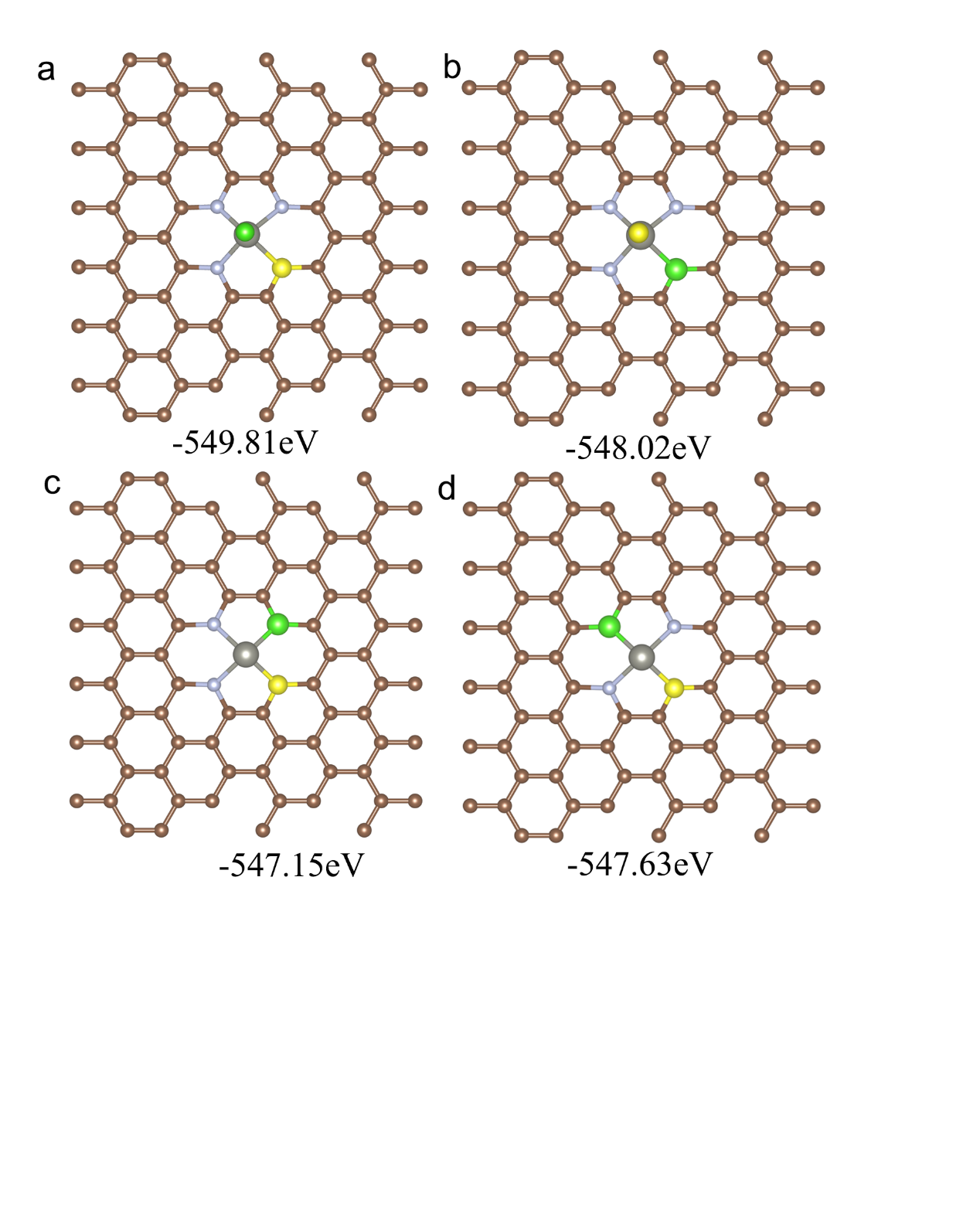


**Figure S29.** Optimized structural models of (a) ZnN_3_S_1_Cl/C, (b) ZnN_3_Cl_1_S/C, (c) ZnN_2_SCl/C-1, and (d) ZnN_2_SCl/C-2.


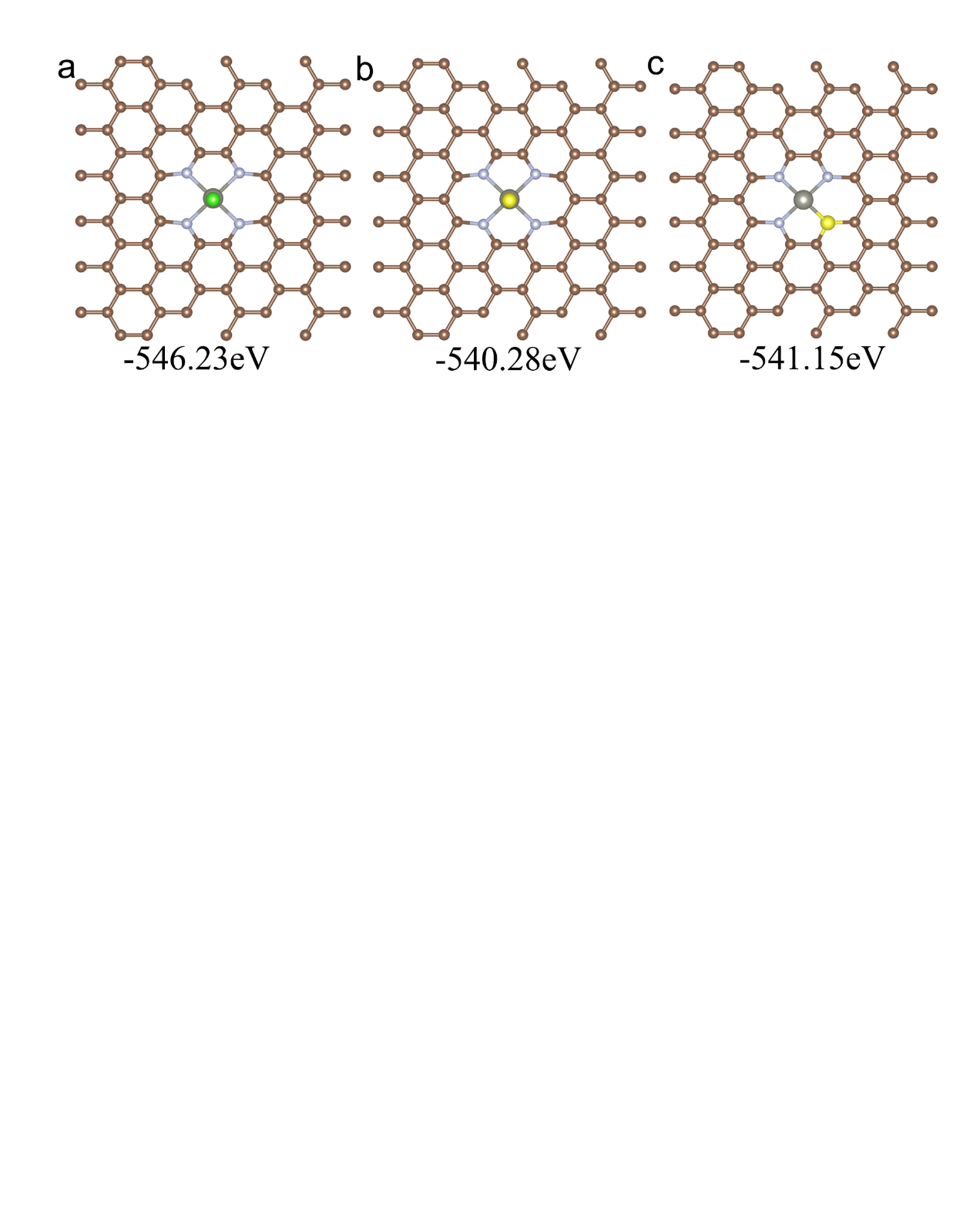


**Figure S30.** Optimized structural models of (a) ZnN_4_Cl/C, (b) ZnN_4_S/C, and (c) ZnN_3_S_1_/C.


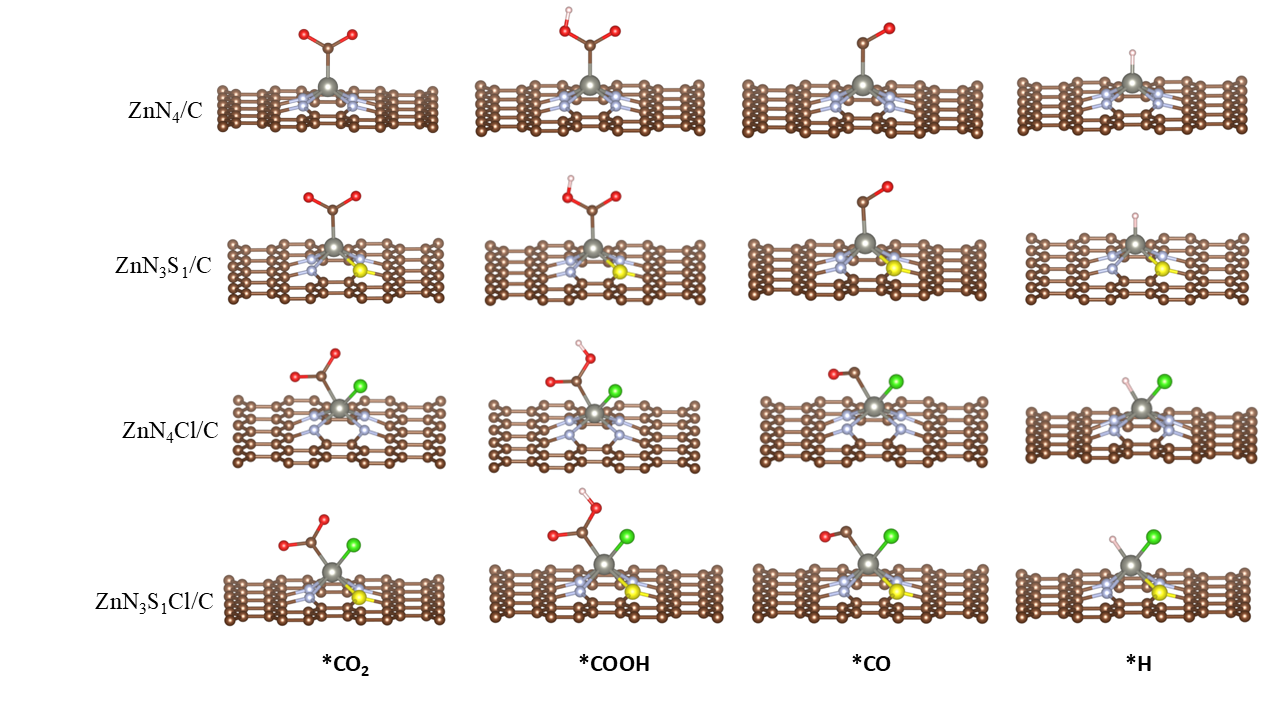


**Figure S31.** Model diagram of catalyst binding with intermediate states in ECR to CO progress.

**Table S1**. Structural parameters extracted from XAFS fitting data.

| sample | Path | CN | R (Å) | σ^2^(10^-3^ Å^2^) | ΔE(eV) | R factor |
| --- | --- | --- | --- | --- | --- | --- |
| ZnN_4_/C | Zn-N | 4.1±0.4 | 1.98±0.01 | 5.2±1.5 | 2.3±1.4 | 0.009 |
| ZnN_3_S_1_Cl/C | Zn-N  Zn-S  Zn-Cl | 3.2±1.7  1.0±0.5  0.97±0.2 | 1.94±0.03  2.24±0.04  2.31±0.02 | 4.6±0.9  5.6±1.3  5.7±1.1 | 5.1±2.1 | 0.01 |

CN: coordination numbers; R: bond distance; σ^2^: Debye–Waller factors; ΔE: the inner potential correction; R factor: goodness of fit.

**Table S2.** Performance comparison of different catalysts.

| Catalysts | Electrolyte | Overpo-tential  (mV) | Current density  (mA cm^-2^) | Maximum FE_CO_ (%) | Stability (h) | Refs |
| --- | --- | --- | --- | --- | --- | --- |
| ZnN_3_S_1_Cl/C | 0.5 M KHCO_3_ | 550 | 7 | 99.02 | 240 | This work |
| ZnN_4_S_1_/P-HC | 0.1 M KHCO_3_ | 600 | 9 | ~ 100 | 30 | ^1^ |
| NiZn-N_6_-C | 0.5 M KHCO_3_ | 800 | 20 | 99 | 27 | ^2^ |
| ZnN/CNO | 0.5 M KHCO_3_ | 470 | 15 | 97 | 200 | ^3^ |
| H-3DOM-ZnN_4_/P-C | 0.1 M KHCO_3_ | 600 | 20 | ~ 100 | 30 | ^4^ |
| Zn^δ+^-NC | 0.5 M KHCO_3_ | 470 | 0.7 | 99 | 12 | ^5^ |
| Fe-N/P-C | 0.5 M KHCO_3_ | 450 | 2 | 98 | 24 | ^6^ |
| Ni-N-C | 0.5 M KHCO_3_ | 700 | 37.8 | 98.5 | 60 | ^7^ |
| FNC-SnOF | 0.1 M KHCO_3_ | 750 | 8 | 95.2 | 28 | ^8^ |
| MoFe-N-C | 0.5 M KHCO_3_ | 600 | 13 | 95.96 | 100 | ^9^ |
| Ni-N_3_-NCNFs | 0.5 M KHCO_3_ | 720 | 30 | 96 | 100 | ^10^ |
| Fe-N_5_/DPCF | 0.5 M KHCO_3_ | 500 | 9.4 | 93.1 | 25 | ^11^ |
| Co-N_2_ | 0.5 M KHCO_3_ | 680 | 18.1 | 95 | 60 | ^12^ |
| Pb-N_2_SV | 0.5 M KHCO_3_ | 470 | 6 | 97.3 | 33 | ^13^ |
| Cu SAs/NC | 0.1 M KHCO_3_ | 700 | 4 | 92 | 60 | ^14^ |
| Co-Tpy-C | 0.5 M NaClO_4_ | 800 | 6.2 | 98 | 24 | ^15^ |
| CoSA/HCNFs | 0.1 M KHCO_3_ | 600 | 22.5 | 97 | 50 | ^16^ |
| Mn-NO/CNs | 0.5 M KHCO_3_ | 460 | 3 | 96 | 70 | ^17^ |

**Table S3.** Performance in Flow-cell/MEA comparison of different catalysts.

| Catalysts | Electrolyte | Current density (mA cm^-2^) | Stability (h) | Refers |
| --- | --- | --- | --- | --- |
| ZnN_3_S_1_Cl/C | 1M KOH | 45 | 12 | **This work** |
| Bi₂O₂NCN | 0.5 M KHCO_3_ | 100 | 20 | ^18^ |
| Cu_2_O-Cd(OH)_2_ | 0.5 M KHCO_3_ | 300 | 24 | ^19^ |
| CuO/Cu_SA2_ | 1 M KOH | 400 | 5 | ^20^ |
| i-In_2_S_3_/S-C | 0.5 M KHCO_3_ | 24 | 75 | ^21^ |
| CeO_2_/AgNWs | 1 M KOH | 258 | 10 | ^22^ |
| Cu-GB | 1 M KOH | 150 | 60 | ^23^ |
| AuCu24-dppp/GED | 1 M KOH | 200 | 100 | ^24^ |
| COF-366-OH-Cu | 1 M KOH | 359 | 1.5 | ^25^ |
| NiSn-APC | 0.5 M KHCO_3_ | 23 | 23 | ^26^ |

**References**

[1] C. Hu, Y. Zhang, A. Hu, Y. Wang, X. Wei, K. Shen, L. Chen, Y. Li, *Adv. Mater.* **2023**, *35*, 2209298.

[2] Y. Li, B. Wei, M. Zhu, J. Chen, Q. Jiang, B. Yang, Y. Hou, L. Lei, Z. Li, R. Zhang, Y. Lu, *Adv. Mater.* **2021**, *33*, 2102212.

[3] Z. Hao, J. Chen, D. Zhang, L. Zheng, Y. Li, Z. Yin, G. He, L. Jiao, Z. Wen, X.-J. Lv, *Sci. Bull.* **2021**, *66*, 1649.

[4] C. Hu, W. Yao, X. Yang, K. Shen, L. Chen, Y. Li, *Adv. Sci.* **2024**, *11*, 2306095.

[5] S. Li, S. Zhao, X. Lu, M. Ceccato, X. Hu, A. Roldan, J. Catalano, M. Liu, T. Skrydstrup, K. Daasbjerg, *Angew Chem. Int. Ed.* **2021**, *60*, 22826.

[6] K. Li, S. Zhang, X. Zhang, S. Liu, H. Jiang, T. Jiang, C. Shen, Y. Yu, W. Chen, *Nano Lett.* **2022**, *22*, 1557.

[7] Y. Zhou, Q. Zhou, H. Liu, W. Xu, Z. Wang, S. Qiao, H. Ding, D. Chen, J. Zhu, Z. Qi, X. Wu, Q. He, L. Song, *Nat. Commun.* **2023**, *14*, 3776.

[8] W. Ni, Y. Gao, Y. Lin, C. Ma, X. Guo, S. Wang, S. Zhang, *ACS Catal.* **2021**, *11*, 5212.

[9] Z. Jin, M. Yang, Y. Dong, X. Ma, Y. Wang, J. Wu, J. Fan, D. Wang, R. Xi, X. Zhao, T. Xu, J. Zhao, L. Zhang, D. J. Singh, W. Zheng, X. Cui, *Nano-Micro Lett.* **2024**, *16*, 4.

[10] W. Zheng, Y. Wang, L. Shuai, X. Wang, F. He, C. Lei, Z. Li, B. Yang, L. Lei, C. Yuan, M. Qiu, Y. Hou, X. Feng, *Adv. Funct. Mater.* **2021**, *31*, 2008146.

[11] Z. Li, J. Jiang, X. Liu, Z. Zhu, J. Wang, Q. He, Q. Kong, X. Niu, J. S. Chen, J. Wang, R. Wu, *Small* **2022**, *18*, 2203495.

[12] X. Wang, Z. Chen, X. Zhao, T. Yao, W. Chen, R. You, C. Zhao, G. Wu, J. Wang, W. Huang, J. Yang, X. Hong, S. Wei, Y. Wu, Y. Li, *Angew. Chem. Int. Ed.* **2018**, *57*, 1944.

[13] S. Zhou, W. Wei, X. Cai, D. Ma, S. Wang, X. Li, Q. Zhu, *Adv. Funct. Mater.* **2024**, *34*, 2311422.

[14] F. Yang, X. Mao, M. Ma, C. Jiang, P. Zhang, J. Wang, Q. Deng, Z. Zeng, S. Deng, *Carbon* **2020**, *168*, 528.

[15] P. Hou, W. Song, X. Wang, Z. Hu, P. Kang, *Small* **2020**, *16*, 2001896.

[16] H. Yang, Q. Lin, Y. Wu, G. Li, Q. Hu, X. Chai, X. Ren, Q. Zhang, J. Liu, C. He, *Nano Energy* **2020**, *70*, 104454.

[17] W. Dong, N. Zhang, S. Li, S. Min, J. Peng, W. Liu, D. Zhan, H. Bai, *J. Mater. Chem. A.* **2022**, *10*, 10892.

[18] Q. Li, B. Gao, X. Zheng, X. Liu, X. Cui, M. Cheng, X. Yan, H. Liu, H. Yang, W. Kong, Z. Wang, W. Wu, Q. Xu, *Appl. Catal. B: Environ. and Energy* **2025**, *368*, 125133.

[19] J. Ma, M. Chen, Q. Yuan, X. Liu, X. Yin, B. Wang, J. Xu, H. He, *Small* **2025**, DOI 10.1002/smll.202501383.

[20] Z. Xu, X. Zhang, Q. Wang, *Chem. Eng. J.* **2024**, *492*, 152164.

[21] F. Chen, J. Fu, L. Ding, X. Lu, Z. Jiang, X. Zhang, J.-S. Hu, *Chin. J. Catal.* **2025**, *71*, 138.

[22] Y.-H. Yu, X. Cui, Y.-M. Hong, G.-W. Qin, S. Li, *Appl. Catal. B: Environ. and Energy* **2025**, *373*, 125352.

[23] L. Wang, X. Yao, H. Fruehwald, D. Akhmetzyanov, M. Hanson, N. Chen, R. Smith, C. V. Singh, Z. Tan, Y. A. Wu, *Adv. Energy Mater.* **2025**, *15*, 2402636.

[24] X. Ma, C. Fang, M. Ding, Y. Zuo, X. Sun, S. Wang, *Angew. Chem. Int. Ed.* **2025**, *64*, e202500191.

[25] Y. Yang, Y. Wang, L. Dong, Q. Li, L. Zhang, J. Zhou, S. Sun, H. Ding, Y. Chen, S. Li, Y. Lan, *Adv. Mater.* **2022**, *34*, 2206706.

[26] W. Xie, H. Li, G. Cui, J. Li, Y. Song, S. Li, X. Zhang, J. Y. Lee, M. Shao, M. Wei, *Angew. Chem. Int. Ed.* **2021**, *60*, 7382.
